# The Co-factor of LIM Domains (CLIM/LDB/NLI) Maintains Basal Mammary Epithelial Stem Cells and Promotes Breast Tumorigenesis

**DOI:** 10.1371/journal.pgen.1004520

**Published:** 2014-07-31

**Authors:** Michael L. Salmans, Zhengquan Yu, Kazuhide Watanabe, Eric Cam, Peng Sun, Padhraic Smyth, Xing Dai, Bogi Andersen

**Affiliations:** 1 Department of Biological Chemistry, University of California, Irvine, Irvine, California, United States of America; 2 Institute for Genomics and Bioinformatics, University of California, Irvine, Irvine, California, United States of America; 3 State Key Laboratories for AgroBiotechnology, College of Biological Sciences, China Agricultural University, Beijing, PR China; 4 Department of Computer Science, University of California, Irvine, Irvine, California, United States of America; 5 Department of Medicine, University of California, Irvine, Irvine, California, United States of America; National Cancer Institute, United States of America

## Abstract

Mammary gland branching morphogenesis and ductal homeostasis relies on mammary stem cell function for the maintenance of basal and luminal cell compartments. The mechanisms of transcriptional regulation of the basal cell compartment are currently unknown. We explored these mechanisms in the basal cell compartment and identified the Co-factor of LIM domains (CLIM/LDB/NLI) as a transcriptional regulator that maintains these cells. Clims act within the basal cell compartment to promote branching morphogenesis by maintaining the number and proliferative potential of basal mammary epithelial stem cells. Clim2, in a complex with LMO4, supports mammary stem cells by directly targeting the *Fgfr2* promoter in basal cells to increase its expression. Strikingly, Clims also coordinate basal-specific transcriptional programs to preserve luminal cell identity. These basal-derived cues inhibit epidermis-like differentiation of the luminal cell compartment and enhance the expression of luminal cell-specific oncogenes ErbB2 and ErbB3. Consistently, basal-expressed Clims promote the initiation and progression of breast cancer in the MMTV-PyMT tumor model, and the Clim-regulated branching morphogenesis gene network is a prognostic indicator of poor breast cancer outcome in humans.

## Author Summary

Recent advancements in mammary gland biology demonstrate conflicting models in maintenance of basal and luminal cell compartments by either unipotent or bipotent mammary stem cells. However, the molecular mechanisms underlying control of the basal cell compartment, including stem cells, remain poorly understood. Here we explore the currently unknown transcriptional mechanisms of basal stem cell (BSC) maintenance, in addition to addressing the role of the basal cell compartment in preserving luminal cell fate and promoting development of human breast tumors of luminal origin. We discover a novel function for the Co-factor of LIM domains (Clim) transcriptional regulator in promoting mammary gland branching morphogenesis and breast tumorigenesis through maintenance of the basal stem cell population. The transcriptional networks coordinated by Clims in basal mammary epithelial cells also preserve the identity of luminal epithelial cells, demonstrating a crosstalk between these two cellular compartments. Furthermore, we correlate developmental gene expression data with human breast cancer to investigate the role of developmental pathways during the initiation and progression of breast cancer. The gene regulatory networks identified during development, including those specifically coordinated by Clims, correlate with breast cancer patient outcome, suggesting these genes play an important role in the progression of breast cancer.

## Introduction

Mouse mammary gland morphogenesis begins during mid-gestation with the development of two bilateral epithelial ridges along the ventral epidermis that form invasive, multipotent stem cell-enriched placodes migrating into the underlying mesenchyme, later branching to form a rudimentary ductal tree by birth. The structure remains relatively quiescent until hormonal stimuli at puberty initiate the formation of stem cell-enriched terminal end buds (TEBs) that rapidly proliferate and invade into the mammary fat pad, frequently bifurcating to generate a ductal network. The resulting ducts consist of luminal epithelial cells surrounded by a basal myoepithelial cell layer. Basal and luminal cells communicate signals to each other through paracrine and direct cell-cell interactions to regulate proper morphogenesis [1], [2]; the nature of these complex cell-cell interactions remains to be fully defined.

Mammary stem cells (MaSCs) coordinate ductal morphogenesis and homeostasis of the luminal and basal cell compartments in the adult mammary gland. Two models have been proposed for the function of MaSCs in the mammary gland: either committed unipotent luminal and basal epithelial stem cells maintain their respective compartment [3], or bipotent MaSCs in the basal cell compartment give rise to both lineages [4]. CD49f^Hi^CD29^Hi^CD24^+^ basal epithelial cells maintain a small population of basal stem cells (BSCs) with the potential to regenerate a functional mammary gland [5], [6], while luminal stem cells (LSCs), enriched in the CD49f^L^°CD29^L^°CD24^+^ luminal epithelial cell population, maintain unipotent potential to preserve the luminal cell population [3], [4] and cannot regenerate the mammary gland. Transcription factors that control the maintenance of stem cells and lineage specification along the mammary epithelial cell (MEC) hierarchy are best characterized in the luminal cell compartment [7]. However, the current knowledge of transcriptional regulation of BSCs and their differentiation is limited.

The LIM domain, a tandem zinc finger motif that serves as an interface for protein-protein interactions, is found in a variety protein families, including the LIM-homeodomain (Lhx) and LIM-only (LMO) transcription factors [8]. Originally discovered as a co-activator of Lhx and LMO [9]–[12], the co-factor of LIM domains (CLIM/LDB/NLI) coordinates the organization of transcriptional complexes through two key regulatory domains: the amino-terminal homodimerization domain and the carboxy-terminal LIM-interacting domain (LID) [9], [13], [14]. Additionally, Clims can associate with other DNA-binding proteins, including GATA, bHLH, and Otx family members [15], [16]. It is through these higher order transcriptional complexes that Clims mediate enhancer-promoter interactions to influence gene regulation [17]–[19]. The Clim family consists of two highly conserved members: the ubiquitously expressed *Clim2* (*Ldb1*/*Nli*), and the more regionally expressed *Clim1* (*Ldb2*) [10]. Germline deletion of *Clim2* in the mouse causes embryonic lethality by day 9.5 [20], while *Clim1* knockout mice display no developmental defects (as indicated by the Mouse Genome Informatics website) possibly due to compensatory effects by Clim2. The function of Clims in mammary gland development has not been investigated. However, in breast cancer, Clim expression correlates with estrogen receptor (ER) positivity where Clims are involved in coordinating transcriptional networks through ERα [21].

Because of the lethality of germline *Clim2* deletion and the possible overlap in function between Clim1 and Clim2, we investigated the role of Clims in the mammary gland using the Keratin 14 (K14) promoter to express a dominant-negative Clim (DN-Clim), consisting of the highly conserved LID fused to a nuclear localization signal and Myc-tag [22], directing its expression to basal MECs. The DN-Clim molecule, which targets all Clim family members, interacts with LIM domain proteins through the LID, but lacks the dimerization domain essential for coordinating enhancer-promoter interactions [18], thereby inhibiting Clim-mediated transcriptional regulation. Using the K14-DN-Clim mouse model we discovered novel transcriptional regulatory programs coordinated by Clims in the basal cells of the mammary gland that promote branching morphogenesis through the maintenance of BSCs, in addition to controlling basal cell-derived transcriptional programs that preserve luminal cell identity. Transcriptional networks within the stem cell-enriched TEB and differentiated duct correlate with human breast cancer gene expression and patient outcome in an opposing fashion; a significant proportion of these networks are regulated by Clims. Furthermore, Clim-mediated transcriptional machinery in the basal cell population permits tumor initiation and progression in the MMTV-Polyoma Middle T (PyMT) breast cancer model. Altogether, these data describe novel mechanisms of transcriptional regulation in BSCs and their role in breast cancer.

## Results

### Clims promote mammary gland branching morphogenesis

**Figure 1:**
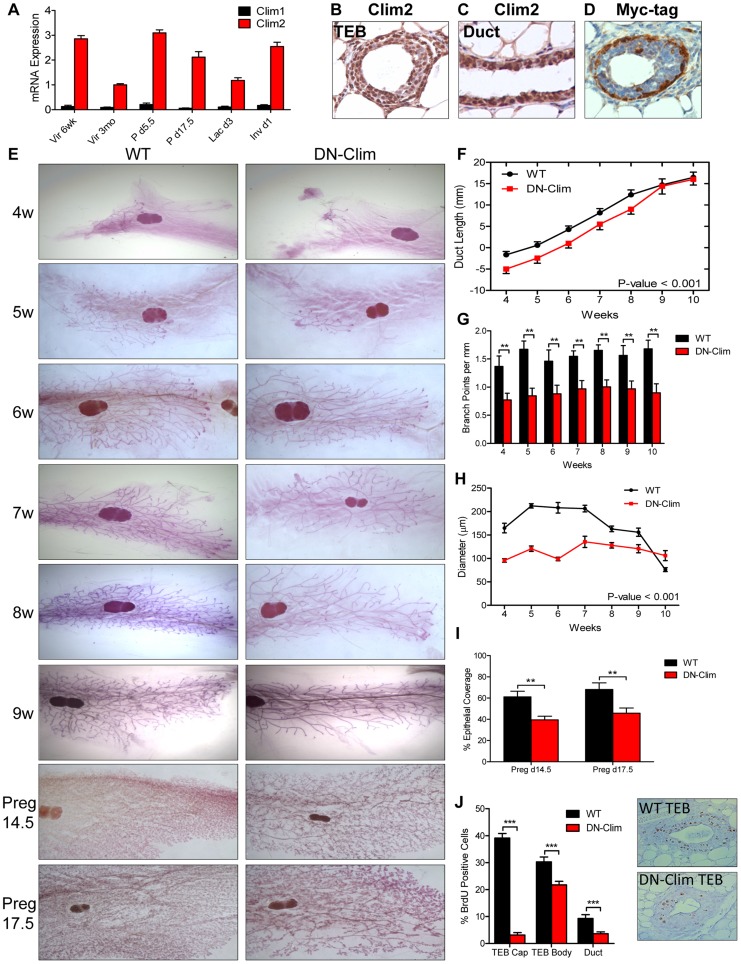
Clims promote branching morphogenesis and proliferation. (A) Clim2 mRNA is predominantly expressed over Clim1 throughout mammary gland development. Gene expression is normalized to 18s mRNA. (B–C) IHC showing expression of Clim2 in the (B) TEB and (C) duct in mammary glands from 6 week old mice. Clim2 expression is observed in basal and luminal cells, with slightly higher expression in basal cells. (D) IHC detecting the Myc-tag of DN-Clim demonstrates expression in the K14-positive basal layer of mammary glands from 6 week old mice. (E) Whole mount analysis of mammary glands throughout branching morphogenesis and pregnancy demonstrates defective branching morphogenesis and TEB maintenance in DN-Clim mammary glands. Images are representative of at least three mice per time point. (F) Mean ductal penetration of the three longest ducts throughout branching morphogenesis indicates a delay in DN-Clim ductal penetration. Data are normalized using the center of the lymph node as the central reference point. P-value derived from repeated measures ANOVA. (G) Mean number of branch points for the three longest ducts normalized to the length of the duct (mm) indicates reduced branching frequency in DN-Clim mammary glands. ** P-value<0.01 determined by Student's t-test. (H) TEB diameter (µm) is reduced in DN-Clim mammary glands throughout branching morphogenesis. P-value derived from repeated measures ANOVA. (I) Proportion of pregnancy stage mammary fat pad filled with epithelial tissue demonstrates a reduced epithelial density in DN-Clim mammary glands. ** p-value<0.01 determined by Student's t-test. (J) Proportion of BrdU positive cells in TEB (cap and body cells) and duct cells is reduced in DN-Clim mammary glands. Representative images of the TEB are shown. *** p-value<0.01 determined by Student's t-test. Quantification for A, F-J represent mean ± SEM for at least three mice.

Among the two Clim family members, *Clim2* is preferentially expressed throughout mammary development (Figure 1A), localized to basal and luminal MECs (Figure 1B–C, S1A). To study the role of Clims in MECs we used the K14-DN-Clim mouse model, targeting DN-Clim expression to the K14-positive basal MECs, as indicated by Myc-tagged DN-Clim expression (Figure 1D). DN-Clim is only expressed in basal cells of ducts and TEB cap cells with rare expression in the TEB body cells, consistent with K14 expression in a subset of these cells [23].

Abnormalities in branching morphogenesis of DN-Clim mammary glands appear as early as 4 weeks of age and continue through adulthood and late pregnancy (Figure 1E), featuring delayed ductal elongation, reduced branching frequency, and smaller TEBs (Figure 1F–H). While the delayed ductal penetration dissipates by early adulthood, the reduced branching frequency and smaller TEBs persist throughout development. Apart from the reduction in size, the TEBs of DN-Clim mammary glands appear to be morphologically normal. While branching morphogenesis is typically completed by week 10, as denoted by the absence of TEBs, small DN-Clim TEBs persist beyond 10 weeks of age (Figure 1H). Collectively, these data demonstrate an important role for basal cell-expressed Clims in mammary gland branching morphogenesis through enhancement of ductal penetration, branching frequency, and TEB size.

During pregnancy, DN-Clim mammary glands at day 14.5 and 17.5 exhibit reduced epithelial density (Figure 1E and 1I); however, this could be a result of the low ductal density prior to pregnancy, rather than defective side branching and alveologenesis. Additionally, while DN-Clim females produce litters comparable in size to normal mice, they can only support a limited number of pups beyond postnatal day 2 (Figure S1B), likely due to decreased milk availability from the underdeveloped mammary epithelial tree, as the surviving pups from DN-Clim females grow at a normal rate (Figure S1C) and wild type lactating females can support full litters derived from DN-Clim females. Thus, Clims are required for a fully functional lactating mammary gland.

### Clims maintain cell proliferation during branching morphogenesis

To determine the cellular mechanism for impaired mammary gland development in DN-Clim mice we measured bromodeoxyuridine (BrdU) incorporation in proliferating cells, observing significant decreases in BrdU^+^ cells in DN-Clim TEB and ducts (Figure 1J). Consistent with the expression pattern of DN-Clim in TEB cap cells, we observed the most drastic reduction of proliferation in the cap cells, while the body cells exhibit only a moderate, yet significant, decrease in proliferation. The fact that TEB body cells, which rarely express DN-Clim, have reduced proliferative potential suggests a non-autonomous mechanism of Clim-mediated cell proliferation through signals that originate from the cap cells. Thus, Clims promote proliferation in both the TEB and duct during branching morphogenesis.

### Gene sets differentially expressed in the TEB and duct represent distinct developmental functions during branching morphogenesis and are differentially expressed in breast cancer subtypes

The current knowledge of the molecular mechanisms that regulate branching morphogenesis is limited, especially regarding the transcriptional programs within the stem cell-enriched TEB and differentiated duct structures. To elucidate these transcriptional programs, and specifically those controlled by Clims, we profiled gene expression in TEB and duct cells from wild type (WT) and DN-Clim mice, using laser capture microdissection to isolate these structures from four, six, eight, and ten week old mice. Consistent with its preferential expression during branching morphogenesis, *Clim2* was expressed higher than *Clim1* in both the TEB and duct cells (Figure S2A).

**Figure 2:**
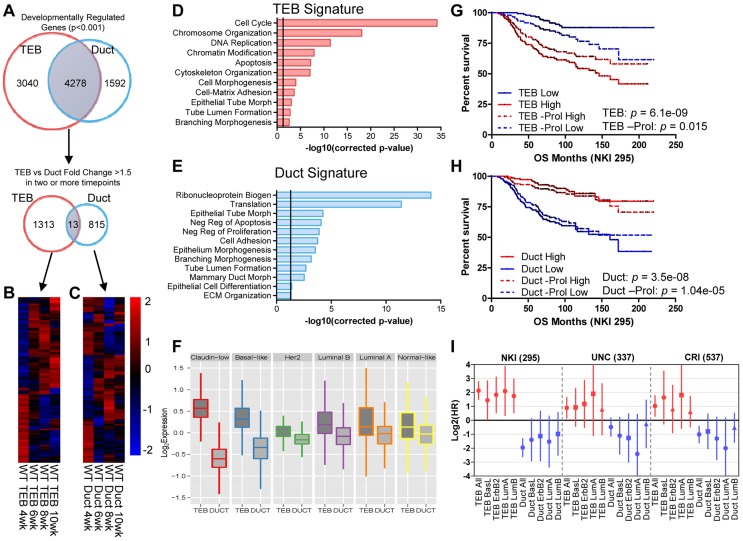
Developmental TEB and duct gene signatures derived from time course expression profiling predict breast cancer prognosis. (A) Identification of developmentally regulated genes with the BETR algorithm in TEB and duct cells over the time course of branching morphogenesis (4, 6, 8, and 10 weeks). TEB and duct signatures were further refined to by selecting genes with at least 1.5-fold expression change in at least two time points for each cell type. (B–C) Heat map of resulting (B) 1,313 TEB and (C) 815 duct gene signatures expressed at least 1.5-fold or greater in their respective cell type in at least two time points. (D–E) Biological processes associated with (D) TEB and (E) duct gene signatures. The TEB and duct signatures represent a proliferation and differentiation signature, respectively. Vertical line represents p-value at 0.05. (F) TEB and Duct signatures are differentially expressed in the molecular breast cancer subtypes. The TEB and duct signatures are more highly expressed in the poorly- and well-differentiated breast cancer subtypes, respectively. (G–H) Analysis of overall survival (OS) in NKI 295 cohort of patients classified into (G) TEB and (H) duct high and low expression groups based on median expression the respective gene signature, including the effects of removing proliferation genes (-Prol). High expression of the proliferative TEB signature confers poor prognosis, while high expression of the differentiated duct signature confers improved prognosis. P-value derived from Log-rank test. (I) Hazard ratios (HR) derived from overall survival analysis for TEB and duct signatures in the molecular subtypes of breast cancer in three separate data sets demonstrate similar trends observed in G–H. Red samples represent TEB signature and blue samples represent duct. Error bars represent 95% confidence interval; filled circles represent p-value<0.1, squares represent trends with p-value<0.25, and triangles represent non-significant changes with p-value>0.25.

To define developmentally regulated genes over the time course we applied the Bayesian Estimation of Temporal Regulation (BETR) algorithm [24] identifying 8,030 and 6,488 probe sets (7,318 and 5,870 genes) temporally regulated in TEB and duct cells, respectively, with 1,313 TEB and 815 duct genes changing 1.5-fold or greater in two or more time points (Figure 2A–C, Dataset S1 and S2). TEB genes are associated with proliferation, while the duct genes are associated with differentiation (Figure 2D–E). Because the TEB represents a stem cell-enriched population [25], and because of our direct comparison of TEB and duct cells, these gene signatures are representative of mammary stem cell and differentiated MECs, respectively.

The hyperproliferative and invasive nature of the TEB bears similarity to molecular properties observed in aggressive breast and other adenocarcinomas. In addition, poorly differentiated basal-like tumors are more aggressive and possess stem cell-like transcriptional characteristics compared to the differentiated luminal subtypes [26]. This prompted us to determine whether the less differentiated TEB and fully differentiated duct gene signatures can be used as predictors of prognosis in human breast cancer. Utilizing three data sets that profile the transcriptome and classify the intrinsic subtypes of primary human breast tumors [27]–[29] we found the TEB signature is highly expressed in the poorly differentiated claudin-low and basal-like breast cancer subtypes, while the duct signature is more highly expressed in the differentiated luminal subtypes (Figure 2F). High TEB signature expression predicted poor overall survival in breast cancer patients (Figure 2G). An opposite trend was observed with the duct signature (Figure 2H), suggesting the expression of differentiation genes suppresses tumorigenesis. These trends are also observed within the different subtypes of breast cancer (Figure 2I). Even after removal of proliferation genes, which significantly contribute to prognosis prediction [30], the TEB signature retains prognostic relevance, albeit with reduced power (Figure 2G). The duct signature, which is poorly enriched for proliferation genes, was relatively unaffected by removal of these genes (Figure 2H). Thus, these developmental signatures hold prognostic value in breast cancer and may be useful to identify novel genes of interest for understanding the biology of breast cancer in relationship to normal development.

### Clims regulate developmentally associated TEB and duct genes

**Figure 3:**
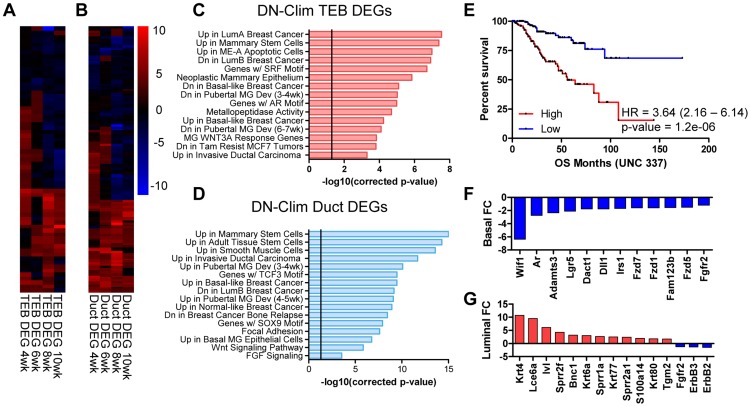
Clims coordinate the expression of developmental gene signatures. (A–B) Heat map of (A) TEB (66 genes) and (B) duct (137 genes) DEGs in the DN-Clim mammary gland with CyberT p-value<0.01 in at least two time points. (C–D) Categories of enriched gene sets from the Molecular Signatures Database that significantly overlap with the (C) TEB and (D) duct DEG sets. Both gene sets are enriched with mammary stem cell and breast cancer genes. Vertical line represents p-value at 0.05. (E) Analysis of overall survival (OS) in UNC 337 cohort of patients classified into high and low expression groups based on median expression of the combined list of DEGs in the DN-Clim TEB and ducts demonstrates that high expression of Clim-regulated genes confers poor prognosis. HR: Hazard Ratio with 95% confidence interval. P-value derived from Log-rank test. (F) Fold change of Wnt-signaling factors in DN-Clim sorted basal cells suggests altered Wnt signaling. Fgfr2 is also downregulated. (G) Increased expression of epidermal differentiation genes in DN-Clim luminal cells suggests a role for basal cells in maintaining luminal cell fate. Luminal cell signaling factors ErbB2/3 and Fgfr2 are also downregulated.

To determine genes regulated by Clims during branching morphogenesis we used the CyberT algorithm [31] to define differentially expressed genes (DEGs) in the DN-Clim TEB and duct: 66 TEB and 137 duct genes were significantly differentially expressed in at least two time points (Figure 3A–B, Dataset S3 and S4), a sizable proportion of which are differentially expressed in both TEB and duct cells (Figure S2B). Each DEG set is significantly enriched with genes from their respective developmental signature (Figure S2C–D), indicating an important role for Clims in regulating branching morphogenesis. Using the Molecular Signature Database [32] to characterize the biological properties of the Clim-regulated TEB and duct gene sets revealed that both have functions in mammary stem cells, pubertal mammary gland development, several breast cancer subtypes, and Wnt-signaling in the mammary gland (Figure 3C–D). Additionally, TEB DEGs participate in metallopeptidase activity and possess serum response factor (SRF) and androgen receptor (AR) transcription factor binding motifs, while duct DEGs are enriched with smooth muscle and mammary basal epithelial cell genes, participate in FGF signaling, and possess TCF3 and SOX9 transcription factor binding motifs. Furthermore, high expression of Clim-regulated genes in breast cancer is an indicator of poor prognosis (Figure 3E). Collectively, these data support the involvement of Clims in gene expression networks important for both stem cell function and breast cancer.

To determine genes differentially expressed in basal and luminal cell compartments we sorted MECs into Lin^−^CD29^Hi^CD24^+^ and Lin^−^CD29^L^°CD24^+^ populations, respectively (Figure S3A–D). Basal and luminal cell purity from WT and DN-Clim MECs was confirmed by qPCR for K8, K14, and the *K14-DN-Clim* transgene expression (Figure S3E–G); as expected, the *K14-DN-Clim* transgene is only expressed in basal cells, and not luminal cells. Basal markers (K5, K14, and smooth muscle actin) and luminal markers (K8, K18, Gata3, estrogen and progesterone receptors) are significantly overexpressed (CyberT, p-value<0.001) in their respective cell types, indicating effective basal and luminal cell separation (Dataset S5). DN-Clim basal and luminal cells differentially express 422 (221 up and 201 down) and 227 (139 up and 88 down) genes with at least 1.5-fold change, respectively (Dataset S6 and S7), indicating both cell and non-cell autonomous mechanisms of gene regulation coordinated by Clims; a significant proportion of basal- and luminal-specific DEGs overlap with the DEGs identified in the TEB and duct populations (Figure S3H–I). Basal-specific DEGs participate in mammary gland morphogenesis processes, including cell proliferation, adhesion, and stem cell properties (Figure 3F and S3J). Interestingly, DEGs in the luminal cell population are enriched with keratinization and squamous epithelial, specifically esophageal and epidermal, differentiation genes (Figure 3G and S3K), suggesting a transepidermal differentiation of the luminal cells in the DN-Clim mammary gland. Thus, Clims coordinate gene expression in the basal cell compartment that ultimately help maintain the identity of luminal cells.

Several genes and pathways related to mammary gland stem cell biology are differentially expressed in the DN-Clim mammary gland throughout the developmental time course and within the basal and luminal cell compartments (Figure 3C–D, and S3J–K). Ontology analyses revealed involvement of Clim-regulated genes in mammary stem cells and the Wnt signaling pathway. Lgr5, a marker of stem cells in CD29^Hi^CD24^+^ basal cells [33], is consistently downregulated in basal cells and both the TEB and duct (Dataset S3, S4, and S6). Additional Clim-regulated genes that have known roles in maintenance of the stem cell enriched TEB and branching morphogenesis are the *Fgfr2* gene [34], [35] and the luminal cell-specific *ErbB2* and *ErbB3* genes (Figure 3G and S4) [36], [37]. Given this data, we hypothesized that Clims promote branching morphogenesis through gene regulation within stem cells of the basal MEC compartment.

### Clims maintain the basal mammary epithelial stem cell population

**Figure 4:**
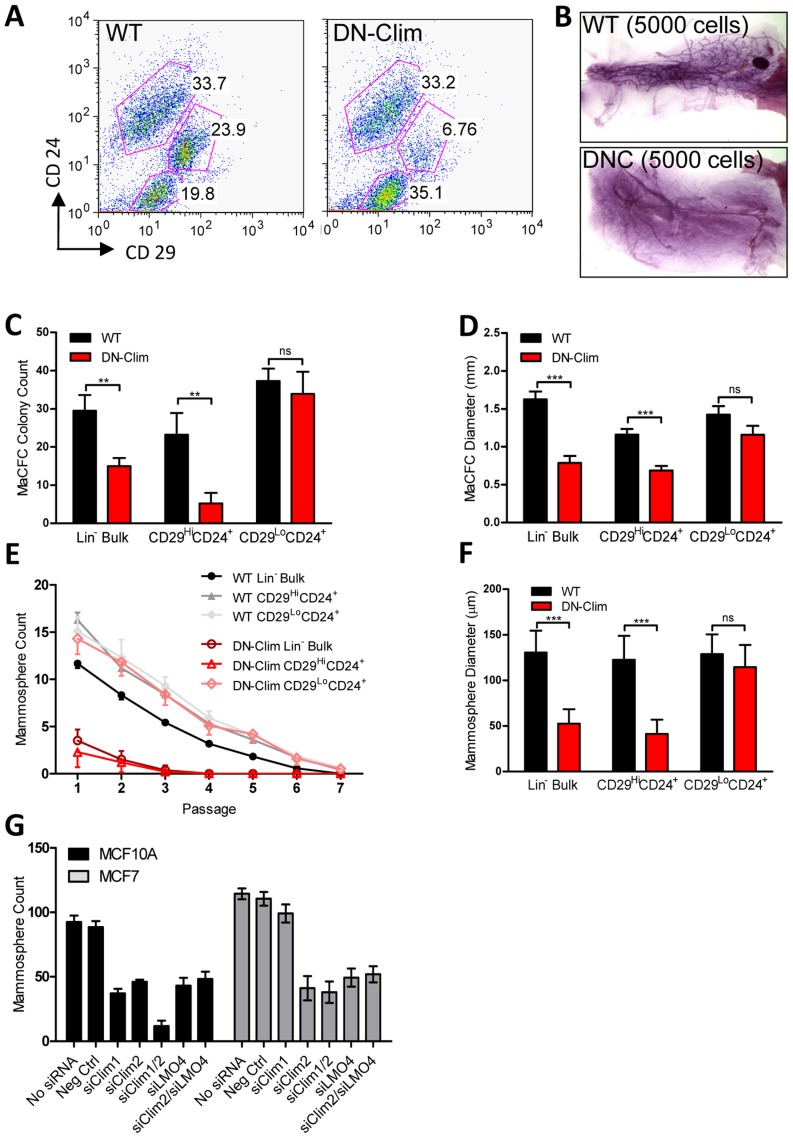
Clims maintain basal mammary epithelial stem cells. (A) Flow cytometry analysis of Lin^−^ MECs with CD29 and CD24 markers reveals decreased CD29^Hi^CD24^+^ basal epithelial cell population. (B) DN-Clim mammary glands are absent of MRUs as demonstrated in representative whole mounts from transplants of CD29^Hi^CD24^+^ BSC-enriched population. See Table 1 for results from limiting dilution analysis. (C) DN-Clim basal cells, and not luminal cells, maintain fewer colony forming units determined by colony-forming cell assays with Lin^−^ bulk and sorted primary mammary epithelial cells. (D) Reduced growth rates in colonies that form from DN-Clim basal cells determined by quantification of colony size for the corresponding MaCFC assays described in (C). (E) DN-Clim basal cells, and not luminal cells, maintain fewer mammosphere forming units determined by mammosphere assays with Lin^−^ bulk and sorted primary mammary epithelial cells. (F) Reduced growth rates in spheres that form from DN-Clim basal cells determined by quantification of mammosphere diameter for the first passage of the mammosphere assays described in (E). (G) Clim1, Clim2 and the Clim interaction factor LMO4 are essential for maintaining stem-like features of MCF10A cells, as determined by mammosphere assays with transient siRNA knockdown of Clim1, Clim2, and LMO4. In MCF7 cells, only Clim2 and LMO4 maintain stem-like features. Data represent mean ± SEM from at least three mice (C–F) or at least three experiments (G). ** p-value<0.01; *** p-value<0.001, ns: not significant.

The reduced size of the stem cell-enriched TEB in DN-Clim mice and the enrichment of mammary gland stem cell genes in our DEG sets, along with previous evidence that Clim2 affects stem cells in the hair follicle [22], prompted us to examine the stem cell populations in the DN-Clim mammary gland. Quantification of the basal and luminal MEC populations revealed a reduction of the CD29^Hi^CD24^+^ BSC-enriched population in the DN-Clim mammary gland (Figure 4A), while the proportion of the CD29^L^°CD24^+^ LSC-enriched population remains unchanged. No differences were observed in the luminal progenitor cell population when using CD61 as a marker for these cells (Figure S5A) [38]. Limiting dilution transplant analysis with CD29^Hi^CD24^+^ cells revealed a nearly complete ablation of DN-Clim mammary repopulating units (MRU) (Table 1, Figure 4B); only two DN-Clim transplants resulted in mammary outgrowths, both of which developed poorly structured mammary trees (Figure S5B). Thus, Clims are of paramount importance in the maintenance of BSCs.

**Table 1:** Clims maintain the basal mammary epithelial stem cell population.

CD29^Hi^CD24^+^ Basal Cells	MaSC Frequency	
	Wild Type	DN-Clim
5000	2/2	1/2
1000	2/2	0/2
500	3/3	0/3
100	2/3	1/3
50	3/5	0/5
MRU Frequency (95% CI)	1/69 (1/28 to 1/172)	1/5566 (1/1288 to 1/24,055)
*p-value* (WT vs DN-Clim)	*p* = 3.58e-09	
*p-value* (single-hit Poisson model)	*p* = 0.748	*p* = 0.222

Mammary colony forming cell (MaCFC) and mammosphere functional assays confirmed the depleted basal stem/progenitor cell population. Unsorted Lin^−^ bulk and sorted CD29^Hi^CD24^+^Lin^−^ DN-Clim primary epithelial cells have fewer MaCFCs and form smaller colonies, while a slight and insignificant decrease in MaCFCs is observed in the sorted CD29^L^°CD24^+^Lin^−^ population (Figure 4C–D). Consistently, Lin^−^ bulk and CD29^Hi^CD24^+^Lin^−^ sorted DN-Clim primary mouse MECs grown in suspension produced significantly fewer and smaller mammospheres (Figure 4E–F), while CD29^L^°CD24^+^Lin^−^ cells exhibit comparable sphere forming efficiency to WT cells. Serial passaging of the mammospheres every seven days resulted in enhanced depletion rates of Lin^−^ bulk and CD29^Hi^CD24^+^Lin^−^ DN-Clim stem/progenitor cells. Collectively, these results suggest Clims maintain the number and proliferative potential of the basal stem/progenitor cell population.

To further confirm the requirement of Clims in promoting stem-like features, Clim1 and Clim2 were knocked down by siRNA, both independently and together, in the MCF10A and MCF7 mammary epithelial cell lines (Figure S5C–D). Clim2 knockdown resulted in significantly reduced mammosphere forming efficiency in both cell lines, while Clim1 knockdown resulted in significant decreases in MCF10A cells, but not MCF7 cells (Figure 4G). Consistently, we observed a synergistic decrease in mammosphere formation efficiency in the combined siClim1/2 in MCF10A cells, but not MCF7 cells. These cell type-specific differences in promoting stem-like properties may be attributed to higher levels of Clim1 in MCF10A cells than MCF7 cells. Altogether these data further support the role of Clims in promoting stem-like features of MECs.

Clim2 is known to interact with LMO4 in MECs; LMO4 promotes mammary gland morphogenesis and breast cancer [39]–[42]. To determine whether Clim2 may be acting through LMO4 to maintain the mammary stem/progenitor cell population we transiently knocked down *LMO4* gene expression individually and in conjunction with *Clim2* in MCF10A and MCF7 cells (Figure S5E). We found similar reduction in mammosphere forming efficiency with individual and combined knockdowns (Figure 4G), suggesting these two transcription factors may target similar mechanisms to promote stem cell features, consistent with these two factors forming a tight complex [43]–[45]. Thus, Clim2 may regulate gene expression in these cells through the organization of transcriptional complexes involving LMO4.

### Clim2 regulates *Fgfr2* to maintain BSCs

**Figure 5:**
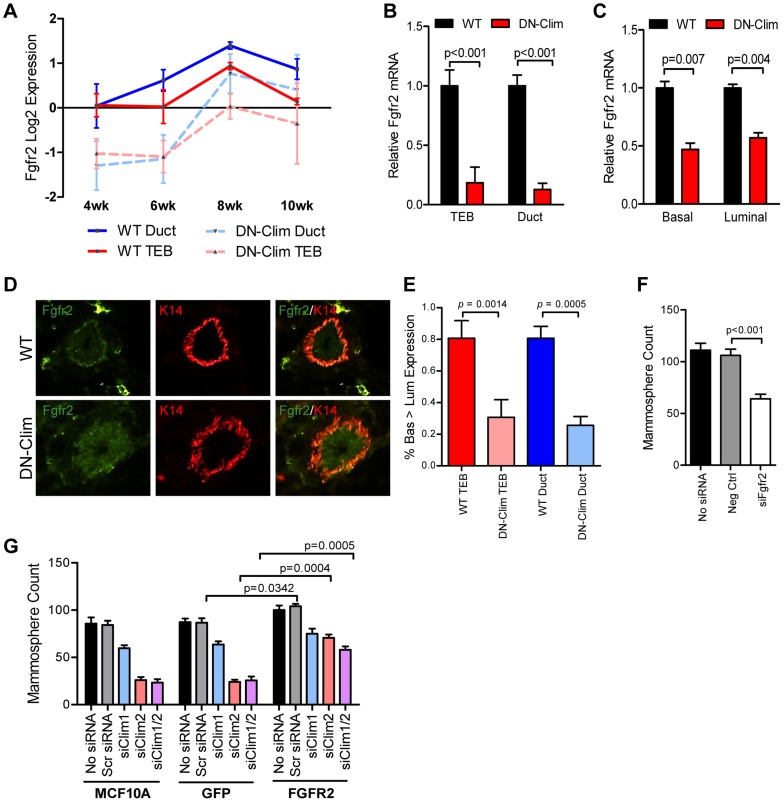
Fgfr2 downregulation in the mammary gland leads to loss of stem/progenitor cell activity. (A) *Fgfr2* is downregulated in TEB and duct cells throughout branching morphogenesis. The data are derived from the time course gene expression microarray analysis. (B) qPCR validation of decreased *Fgfr2* expression in DN-Clim TEB and duct cells. Gene expression is normalized to 18s mRNA. (C) qPCR validation of decreased *Fgfr2* expression in sorted basal and luminal cells. Gene expression is normalized to 18s mRNA. (D) Immunofluorescence analysis of FGFR2 protein expression reveals decreased protein in the basal layer of DN-Clim mammary glands. (E) Semi-quantitative analysis of FGFR2 protein expression in TEB and luminal structures suggests reduced protein levels in basal cells of the DN-Clim mammary gland. WT mammary glands exhibit more protein in basal cells when compared to luminal cells, but DN-Clim mammary glands exhibit equal FGFR2 protein levels in basal and luminal cells. The percentage of TEB and duct structures that exhibit higher FGFR2 protein in basal cells was quantified by counting at least 10 structures from at least 10 frozen sections from three WT and three DN-Clim mammary glands. The data represent the mean ± SEM from three biological replicates. (F) Transient siRNA knockdown of *Fgfr2* in MCF7 reduces sphere forming efficiency in mammosphere assays. (G) Fgfr2 overexpression partially rescues reduced mammosphere forming efficiency upon transient siRNA knockdown of Clims in the MCF10A cell line. Data represent mean ± SEM from at least two littermate mice (A–C, E), or three replicate experiments (F–G).

*Fgfr2* is significantly downregulated in the DN-Clim mammary gland throughout the developmental time course (Figure 5A) as validated by qPCR in the TEB and duct cells from 6 week old mice (Figure 5B). *Fgfr2* expression was also decreased in the sorted basal and luminal cells from DN-Clim mice (Figure 5C). While the decrease in basal cells is likely due to cell autonomous effects of DN-Clim, the decreased *Fgfr2* expression in luminal cells is likely non-cell autonomous as the DN-Clim molecule is not expressed in these cells. Since Fgfr2 is essential for maintaining the BSC population [46], and DN-Clim is localized to the K14-positive basal MECs, we hypothesized that Clims directly regulate the expression of *Fgfr2* to maintain BSCs. Indeed, the FGFR2 protein is decreased in DN-Clim basal cells (Figure 5D–E). Transient knockdown of Fgfr2 in the Fgfr2-high MCF7 cell line impairs mammosphere forming efficiency (Figure 5F), while stable overexpression of Fgfr2 in the Fgfr2-low MCF10A cell line with transient knockdown of Clims partially rescues mammosphere forming efficiency (Figure 5G). These results suggest Clims regulate *Fgfr2* expression to maintain stem-like features.

**Figure 6:**
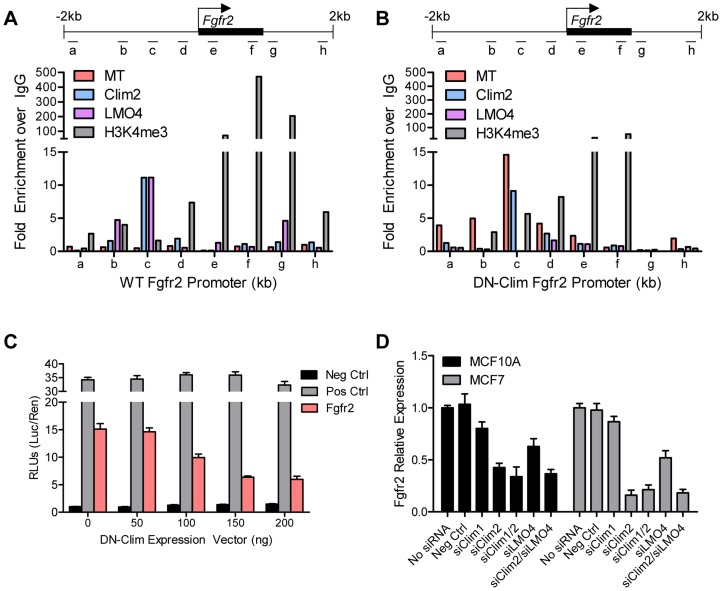
Clims and LMO4 directly target and the *Fgfr2* promoter to induce gene expression. (A–B) ChIP-qPCR assays of Myc-tagged DN-Clim, Clim2, LMO4, and H3K4me3 enrichment at the indicated locations of the ±2 kb surrounding the *Fgfr2* promoter (top panel) in (A) WT and (B) DN-Clim MECs. MECs were collected from three littermate mice then pooled for ChIP-qPCR experiments. The data suggest Clim2 binds approximately 0.5–1.0 kb upstream of the *Fgfr2* TSS. When DN-Clim is bound to this region, LMO4 binding is lost and H3K4me3 levels are decreased. (C) Luciferase assays from MCF10A cells transiently expressing the Clim-binding promoter region of *Fgfr2* in the pGL3-promoter luciferase reporter vector indicate Clim acts as an inducer of *Fgfr2* expression. The DN-Clim expression vector was transiently titrated to demonstrate reduction of luciferase activity with increasing DN-Clim expression. (D) Expression of *Fgfr2* in the MCF10A and MCF7 cells is reduced upon transient siRNA knockdown of Clim1, Clim2, and LMO4. Expression is normalized to GAPDH. Data represent mean ± SEM from three replicate experiments (C–D).

To determine if Clims directly bind the *Fgfr2* promoter, we performed ChIP assays for Clim2, DN-Clim (Myc-tag), H3K4me3 (a marker for actively transcribed genes), and the Clim-interacting partner LMO4, followed by qPCR targeting multiple sites in the 2 kb region surrounding the *Fgfr2* transcriptional start site (TSS). Clim2 binds to a region 538 bp to 708 bp upstream of the TSS in the WT mammary gland, with enriched H3K4me3 surrounding the TSS (Figure 6A), while DN-Clim binds the same region upstream of the TSS in DN-Clim mammary glands, with a drastic reduction in H3K4me3 enrichment (Figure 6B). Additionally, LMO4 is enriched at the same region in WT mammary glands, but is absent in the DN-Clim mammary gland, suggesting a necessity for Clim2 to recruit LMO4 to the promoter. To determine if Clim-binding activates transcription, we cloned the promoter-binding region (433 to 1010 bp upstream of the *Fgfr2* TSS) into the pGL3-promoter vector. In Clim2-positive MCF10A cells, this region of DNA enhances transcription of the luciferase reporter, and DN-Clim inhibits its transcription (Figure 6C). Furthermore, *Fgfr2* expression is downregulated in MCF10A and MCF7cells upon knockdown of Clim1/2 and LMO4 (Figure 6D). Altogether, these data suggest that Clim2 promotes the maintenance of the BSC population by directly binding the *Fgfr2* promoter to drive transcription of the gene.

### Clim2 promotes breast tumorigenesis

**Figure 7:**
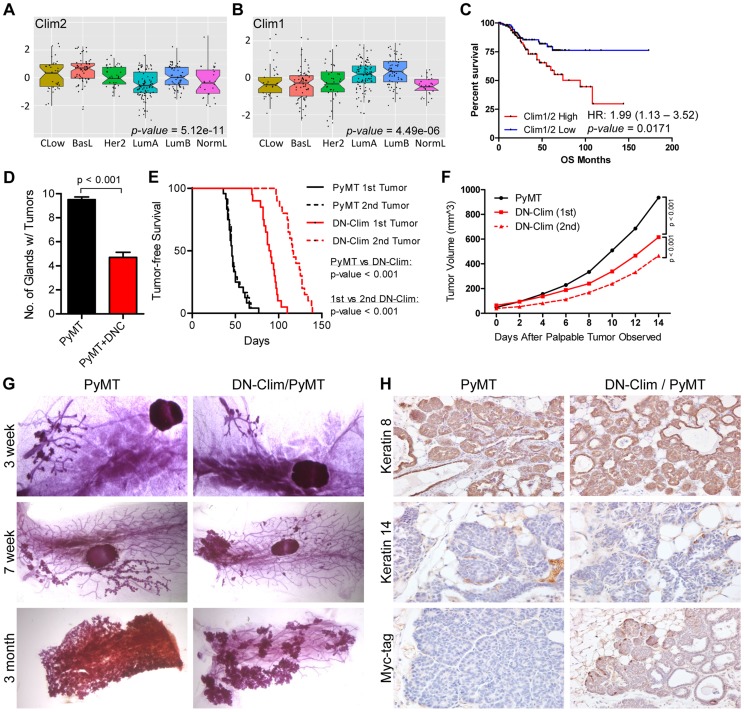
Clims promote the initiation and progression of tumors in the MMTV-PyMT breast cancer mouse model. (A–B) Expression of (A) Clim2 and (B) Clim1 in the molecular subtypes of breast cancer. Clim2 is more highly expressed in less differentiated Claudin-low (CLow) and basal-like (BasL) tumor subtypes and Clim1 is more highly expressed in the differentiated Luminal A and B (LumA and LumB) subtypes. (C) Survival analysis within the UNC 337 cohort of breast cancer patients grouped by median expression of Clim1 and Clim2 demonstrates worse prognosis in patients with highest expression of Clim1/2. (D) DN-Clim prevents tumorigenesis in the PyMT tumor model as determined by quantification of the number of mammary glands with palpable tumors. Data represent mean ± SEM from ten mice of each genotype. (E) DN-Clim expression in the PyMT tumor model significantly delays development of palpable tumors. In PyMT mice, the second palpable tumor is detected within a few days of the first tumor, but when DN-Clim is expressed there is a significant increase in the time to detect the second palpable tumor. P-values derived from Log-rank test. (F) Decreased rate of tumor progression in DN-Clim/PyMT mice. P-values derived from repeated measures ANOVA. (G) Representative whole mounts of PyMT tumors with or without DN-Clim suggest a function for Clim in the tumor initiation stages. The primary tumor was excised from three month old mice before whole mount of the mammary gland. (H) K8, K14, and Myc-tag (DN-Clim) expression in mammary glands from 6 week old PyMT mice with or without DN-Clim. High K8 and low K14 expression demonstrate the luminal subtype features of PyMT tumors. DN-Clim expression is observed in basal cells of the tumor.

The CLIM protein is expressed in ER-positive breast tumors [21], and we show here that the Clim-regulated gene network is associated with poor prognosis in breast cancer (Figure 3E). Furthermore, its interacting partner LMO4 is upregulated in breast cancer and promotes breast tumorigenesis [40], [42], [47], [48]. We examined the expression of Clims in the molecular subtypes of breast cancer and observed highest expression of Clim2 in the less differentiated claudin-low and basal-like breast cancer subtypes, while Clim1 is most highly expressed in the differentiated luminal cell types (Figure 7A–B). Accordingly, Clim2 expression alone predicts poor prognosis in breast cancer, while Clim1 expression displays an opposite trend (Figure S6). The combined expression level of both Clim genes is a strong predictor of poor outcome (Figure 7C), suggesting Clim2 is a more powerful predictor of breast cancer outcome than Clim1.

To determine if Clims play a role in breast tumorigenesis, we bred the *K14-DN-Clim* gene into the MMTV-PyMT mouse model. PyMT mice develop palpable tumors in nearly every mammary gland, whereas DN-Clim/PyMT mice only develop tumors in no more than six mammary glands (Figure 7D). Additionally, DN-Clim/PyMT mice exhibit a significant delay in the development of palpable tumors (Figure 7E) and the growth rate of these tumors is drastically impaired (Figure 7F). While tumor development is rapid and frequent in PyMT mice, with consecutive palpable tumors developing within days of each other, there was a significant delay in the time required for the second palpable DN-Clim/PyMT tumor to develop and the growth rate of the second tumor was significantly slower than the first (Figure 7E–F). Taken together, these data demonstrate that Clim promotes the initiation and progression of breast cancer in the PyMT model.

To determine the stage at which Clim may be promoting breast cancer, we collected whole mounts of mammary glands from as early as 3 weeks to as late as 3 months. Hyperplastic lesions appear in the mammary glands of 3-week-old PyMT and DN-Clim/PyMT mice (Figure 7G). However, by 7 weeks entire arms of the mammary tree display hyperplastic lesions in PyMT mice, while the DN-Clim/PyMT mice only exhibit sparse lesions (Figure 7G). Consistently, after removal of the primary tumor from mammary glands of 3 month old mice we observed a decrease in the area of the epithelial tree covered in tumor tissue. The drastic difference in the number of hyperplastic nodules developing from the mammary tree suggest that Clims act during the early stages of tumorigenesis. IHC staining of 6 week old mammary glands demonstrated that these tumors primarily express K8, and not K14 (Figure 7H), confirming they are luminal tumors [49]. PyMT tumors exhibit a rare population of K14-expressing cells at the leading, basal edge of the tumor, which also express DN-Clim in the DN-Clim/PyMT tumors (Figure 7H). These data demonstrate the necessity of Clim-mediated, basal cell-specific gene expression for robust PyMT-mediated tumorigenesis, and suggest a functional role for Clim-dependent mechanisms in basal MECs during the initiation and progression of luminal breast tumors.

## Discussion

**Figure 8:**
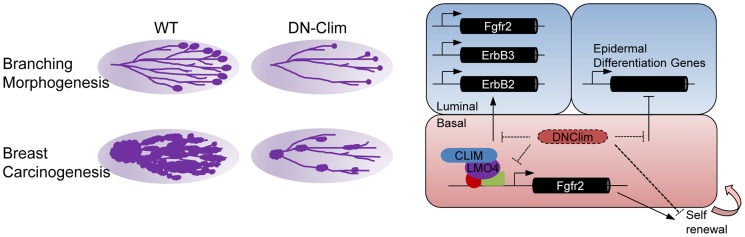
Regulatory roles for Clims in normal development and cancer. Clims promote branching morphogenesis and carcinogenesis in the mammary gland (left). Direct regulation of *Fgfr2* expression by Clims and LMO4 in the basal cell compartment facilitate basal stem cell self-renewal (right). Clims mediate a transcriptional program within the basal cell compartment to promote expression of *Fgfr2, ErbB2*, and *ErbB3* in the luminal cell compartment, in addition to maintaining luminal epithelial cell fate by restricting the expression of epidermal differentiation genes.

Transcriptional networks regulating the development and maintenance of the mammary gland luminal cell compartment are well characterized, with GATA3, Notch1, Elf5, and STAT5a being key factors [38], [50]–[52]. However, transcriptional regulation within the basal cell compartment remains poorly understood. Here, we describe a Clim-driven developmental transcriptional network in the basal cell compartment that maintains the number and proliferative potential of BSCs. Interfering with this transcriptional network leads to defective branching morphogenesis and impaired PyMT-mediated tumorigenesis. In addition, Clim-dependent basal cell-derived transcriptional programs support the expression of key luminal growth factor receptors and maintain the identity of luminal cells (Figure 8).

The widely expressed Clims regulate unique transcriptional programs in diverse cell types. In part, this likely depends on Clim's tissue specific interactions with LIM domains of distinct LHX and LMO family members, such as LMO2 and LHX2 in erythroid cells [12], [53], LHX3 in the neural tube [54], LHX2 in hair follicles [22], [55], [56], and LMO4 in the mammary gland [40]. Despite this diversity in interactions, Clims seem to play a general role in promoting stem cell maintenance as previously shown in intestinal and hair follicle epithelia [22], [57], blood [58], and ES cells [59]. How Clims maintain stem cell features is largely unexplained. We address this question in the mouse mammary gland with the K14-DN-Clim mouse model that targets both Clim family members in the basal cell population. DN-Clim, effectively disrupts Clim/LIM domain-mediated transcriptional complexes *in vivo*, as has been demonstrated in *Drosophila*, Zebrafish, and mouse [22], [60]. Additionally, knockout of *Lhx2*, the Clim-interacting partner in hair follicle stem cells, gives a similar phenotype as expression of DN-Clim [22], [55]. Lacking the dimerization domain, DN-Clim binds LIM domain proteins to inhibit their interactions with endogenous Clims, thereby preventing Clim-mediated looping involved with higher order transcriptional complexes. We still observed DN-Clim at the promoter of the *Fgfr2* gene, indicating that these interactions still allow the binding of LIM domain transcription factors to their target promoters. The N-terminal dimerization domain may have other functions not inhibited by DN-Clim, including possible interactions with other non-LIM domain transcriptional regulators [15], [16] and interactions with the RLIM ubiquitin ligase that leads to degradation of associated LIM domain proteins [61]. DN-Clim has been demonstrated to stabilize nuclear LIM domain proteins [62], which may result in increased levels of these proteins in the basal cells of the mammary gland.

In the mammary gland, several lines of evidence argue that Clims maintain basal stem cells by stimulating expression of *Fgfr2*. Expression of a DN-Clim molecule in the mouse mammary gland and knockdown of Clims in human MECs lead to downregulation of *Fgfr2*. Also, Fgfr2 overexpression can rescue the impaired mammosphere formation after Clim knockdown in MECs. Furthermore, we demonstrate that Clims associate with and regulate the *Fgfr2* promoter in MECs. This model is consistent with work showing that FGF signaling is essential to branching morphogenesis in several epithelial organs, including the drosophila trachea and the mammalian mammary gland, lung, kidney, and salivary gland [63]. FGF signaling promotes mammary embryonic placode formation [64], and Fgfr2 is necessary to maintain the TEB structure and promote primary branching [34], [35]. In addition, Fgfr2 is required for maintenance of the CD29^Hi^CD24^+^ basal cell population and accordingly the maintenance of regenerative BSCs within this population [46]. The defects in branching morphogenesis, basal epithelial cell frequency, and MRU capacity observed in these Fgfr2 transgenic mouse models closely resemble the DN-Clim mammary gland phenotype. Thus, Clim acts immediately upstream of Fgfr2 signaling in the basal cell population through direct regulation of the *Fgfr2* gene to maintain BSCs and promote branching morphogenesis.

Our data suggest that Clim associates with its known mammary gland binding partner LMO4 to regulate the *Fgfr2* gene. We find that LMO4 binds to the same region of the *Fgfr2* promoter, and knockdown of LMO4 also leads to decreased *Fgfr2* gene expression. Furthermore, we find that LMO4 knockdown leads to decreased mammosphere formation in MECs. That these proteins function as a complex is supported by experiments showing that the combined Clim/LMO4 knockdown does not lead to further decrease in mammosphere formation over individual knockdowns. Furthermore, functional studies of LMO4 in the mammary gland demonstrate its role in promoting branching morphogenesis and lobuloalveoar development by sustaining cell proliferation [39], [41]. Altogether, these observations suggest Clims act through LMO4 in the mammary gland to promote BSC maintenance and development.

In addition to regulating the number and replicative potential of BSCs, we find evidence that Clims regulate a basal cell program that maintains luminal cell fate. Since one of the models of stem cell hierarchy in the mammary gland proposes that BSCs give rise to the luminal cells [4], this program may be established cell autonomously in the basal progenitors and persists in the luminal cells. Alternatively, the Clim-controlled program may act through basal-to-luminal cell signaling; there is previous evidence for crosstalk between the basal and luminal compartments through paracrine and cell adhesion-mediated mechanisms [1], [2]. Irrespective of the underlying mechanisms, since DN-Clim is selectively expressed in the basal cell compartment, our gene expression data in basal and luminal cell compartments clearly reveals an effect of basal-specific Clim function on gene expression in luminal cells. In particular, DN-Clim mice exhibit downregulation of key growth factor receptors ErbB2 and ErbB3, and upregulation of several epidermal differentiation genes. These observations demonstrate an essential role for Clims in the basal cell compartment to coordinate gene expression programs in the luminal cell compartment, and for the first time demonstrate the importance of basal cell factors on regulating the expression of key luminal regulators in the EGFR family. These mechanisms may contribute to the abnormal mammary development and decreased breast tumorigenesis in the DN-Clim/PyMT mice.

The role of Clims is not limited to mammary gland development as our work also suggests they act within basal cells during the early stages of tumorigenesis. Expression of DN-Clim within the basal cell compartment significantly impairs the ability of PyMT to initiate neoplastic lesions. In the murine MMTV-PyMT tumor model Fgfr2 is overexpressed in the CD29^Hi^CD24^+^ tumor initiating cell population and is necessary to initiate tumorigenesis in these mice [65]. As Clims are direct regulators of *Fgfr2* in the mammary gland, the reduced tumorigenicity observed in DN-Clim/PyMT mice could in part be explained by Clim-mediated regulation of *Fgfr2* in cancer stem-like cells. The MMTV promoter directs PyMT expression to both luminal and basal cells [66], and while the histology and gene expression patterns group PyMT breast tumors with the luminal subtype of breast cancer, their cell of origin remains unknown [67]. Clims could be promoting early tumorigenesis through one of two scenarios: 1) Clims specifically maintain a basal cell-derived tumor initiating cell, or 2) Clims coordinate the communication of basal-derived tumorigenic signals to the luminal cell compartment, such as promoting the expression of Her2 in luminal cells. These signals would have to occur during the early stages of tumorigenesis when K14-positive basal cells are still present in premalignant lesions [68]. While basal cells have previously been assumed to act as inhibitors of breast cancer progression [69], the restricted tumorigenicity observed in the DN-Clim/PyMT mammary gland suggests basal cells may permit tumorigenesis.

Corresponding to these functional mouse experiments, we observed differential expression of Clims in the molecular subtypes of breast cancer, with high expression leading to poor outcome. It is well known that Clims are expressed in luminal cells and coordinate transcriptional programs through ERα in human breast cancer [21]. However, our K14-DN-Clim mouse model suggests an additional function for Clims during breast tumorigenesis in the ER-negative basal cell population, either through direct regulation of the basal cell or through indirect effects on the luminal cell. Furthermore, we find that a Clim-regulated gene module correlates with poor breast cancer prognosis arguing, for relevance of the mouse experiments for human breast cancer. It is possible that Clims may in part promote breast cancer through activation of *Fgfr2* expression. Deregulation of FGF signaling is observed in breast cancer [70], with *FGFR2* amplification and overexpression observed in 5–10% of human breast cancers, correlating with poor prognosis [71], [72]. In addition, single point mutations in the *FGFR2* gene are associated with increased risk in human breast cancer [73].

In conclusion, we have demonstrated that Clims are a necessary factor in mammary gland branching morphogenesis, maintaining the BSC compartment. Clim2 coordinates with LMO4 to govern these processes through direct regulation of the *Fgfr2* gene. In a breast cancer mouse model Clims act within the basal cells to permit tumorigenesis, possibly through the maintenance of cancer stem-like cells. As Clims are expressed in human breast cancer and correlate with poor differentiation of ER-positive tumors, elucidating Clim targets at a global scale may give insight into the transcriptional mechanisms that maintain primitive cancer cells. The developmental transcriptome of the TEB and duct populations reported will provide a valuable resource for delineating the molecular relationships between development and tumorigenesis, in addition to identifying novel prognostic and therapeutic targets.

## Materials and Methods

### Mouse strains

Generation and maintenance of K14-DN-Clim mice were as previously described [22]. MMTV-PyMT mice (Jackson Labs) were maintained on the K14-DN-Clim mixed background. Fox Chase SCID Beige mice (Charles River) were used as recipients for transplant experiments. All experiments conform to the regulatory guidelines approved by the International Animal Care and Use Committee of the University of California, Irvine.

### Tissue and cell preparation

Mammary glands 4 and 9 were dissected and either whole mounted, paraffin embedded, or frozen in O.C.T. For proliferation assays, mice were injected with BrdU (50 µg/g, Sigma-Aldrich) 4 hours prior to sacrificing. Adult (8 to 12 week) mammary glands for single-cell suspensions were generated according to Stem Cell Technologies protocol. When collecting RNA, the collagenase/hyaluronidase digestion was reduced to 1.5 hours.

### Flow cytometry, sorting, and in vitro culture

Single-cell suspensions were incubated with Propidium Iodide (2 µg/mL, Sigma-Aldrich), CD31-APC, CD45-APC, TER119-APC, CD24-PE (BD Biosciences) and analyzed by flow cytometry on FACSCalibur (BD Biosciences) or sorted on FACSAriaII (BD Biosciences). Bulk primary MECs for in vitro culture were incubated with biotinylated CD31/CD45/TER119 cocktail (Stem Cell Technologies) and magnetically separated to remove lineage cells.

### Cleared fat pad transplantation

FACS-sorted CD29^Hi^CD24^+^ cells were transplanted into cleared fat pads of 3 week old immunocompromised SCID Beige recipients. Mammary glands were evaluated 8 weeks after transplantation. Limiting dilution analysis was performed on the ELDA Web-based tool (http://bioinf.wehi.edu.au/software/elda/).

### Microarray and bioinformatic analysis

RNA was collected from laser capture microdissected (Leica LS-AMD) or FACS sorted MECs with the RNeasy Mini Kit (Qiagen). Gene expression was measured with Affymetrix Mouse Gene 1.0ST array. Data were analyzed with PLIER algorithm. Differential expression was determined with the CyberT algorithm [31] and time course developmental gene expression analysis was performed with the BETR algorithm [24]. Detailed methods of data analysis are described in the supplemental methods accompanying this manuscript.

### Accession numbers

The gene expression microarray data has been submitted to GEO (http://www.ncbi.nlm.nih.gov/geo/) under the accession number GSE57724.

Additional details for the above methods are presented in Text S1, as well as methods for IHC, immunofluorescence, whole mount, ChIP, RT-qPCR, mammosphere and colony forming assays, siRNA transfection, lentiviral expression, luciferase assays and microarray analysis.

## Supporting Information

Dataset S1 List of TEB signature genes. BETR p-values and fold change at each time point is presented for each TEB signature gene. Signature genes have BETR p-value<0.001 and ±1.5 fold change.
(XLSX)

Dataset S2 List of Duct signature genes. BETR p-values and fold change at each time point is presented for each Duct signature gene. Signature genes have BETR p-value<0.001 and ±1.5 fold change.
(XLSX)

Dataset S3 List of DEGs in the DN-Clim TEB. CyberT p-values and fold change at each time point for the TEB are presented. DEGs have p-value<0.01 and ±1.5 fold change in at least two time points. NA: gene did not meet expression criteria in the specified time point.
(XLSX)

Dataset S4 List of DEGs in the DN-Clim Duct. CyberT p-values and fold change at each time point for the Duct are presented. DEGs have p-value<0.01 and ±1.5 fold change in at least two time points. NA: gene did not meet expression criteria in the specified time point.
(XLSX)

Dataset S5 List of DEGs in the WT Basal vs Luminal cell comparison. CyberT p-values and fold change are presented. DEGs have p-value<0.001 and fold change ±1.5.
(XLSX)

Dataset S6 List of DEGs in the DN-Clim basal cell population. CyberT p-values and fold change are presented. DEGs have p-value<0.001 and fold change ±1.5.
(XLSX)

Dataset S7 List of DEGs in the DN-Clim luminal cell population. CyberT p-values and fold change are presented. DEGs have p-value<0.001 and fold change ±1.5.
(XLSX)

Figure S1 Specificity of Clim2 antibody and DN-Clim females fail to support full litters. A) The Clim2 antibody specifically targets the Clim2 protein with no reactivity to Clim1, as determined by western blot on protein lysates from HEK293 cells overexpressing the Clim1 and Clim2 proteins. The Clim1/2 antibody detects Clim1 and Clim2 only in their respective overexpression lysates. Vector Ctrl Lysate = Vector transfected lysate control. (B) Average number of pups per litter from WT and DN-Clim females. DN-Clim mice are unable to support the full litter after postnatal day 2. (C) Growth rate of pups from WT and DN-Clim females. Surviving pups from DN-Clim females grow at a normal rate compared to pups from the WT mother.
(PDF)

Figure S2 Time course analysis of Clim expression and comparison of Clim-regulated genes to TEB and duct genes. (A) Expression of Clim1 and Clim2 from time course analysis of TEB and duct cells. (B) Significant overlap of differentially expressed genes from the DN-Clim TEB and duct. (C–D) DEGs in the DN-Clim (C) TEB and (D) duct are significantly enriched in their respective developmental gene set.
(PDF)

Figure S3 Gene expression profiling in sorted basal and luminal mammary epithelial cells. (A) Selection of live (PI-negative), Lin^−^ (TER119-, CD45-, and CD31-negative) single cells. (B) Gating for basal (Lin^−^CD29^Hi^CD24^+^) and luminal (Lin^−^CD29^L^°CD24^+^) MECs. (C) Post-sort analysis of basal MECs. (D) Post-sort analysis of luminal MECs. APC: Lin markers, PE: CD24, FITC: CD29. (E–F) qPCR validation of (E) Krt14 and (F) Krt8 in sorted cells indicates pure basal and luminal cell populations. (G) qPCR validation of DN-Clim transgene expression confirms expression of DN-Clim in basal cells. (H) DN-Clim basal and (I) DN-Clim luminal DEGs are significantly enriched in the combined list of DN-Clim TEB and Duct DEGs. Ontology analysis of (J) DN-Clim basal DEGs and (K) DN-Clim luminal DEGs. The categories represent top hits from DAVID and the Molecular Signatures Database.
(PDF)

Figure S4 Reduced expression of ErbB2 and ErbB3 receptor tyrosine kinases in the DN-Clim mammary gland. Expression of the (A) ErbB2 and (B) ErbB3 in the time course microarray (left panel), as determined by qPCR in 6 week old laser capture microdissected TEB and duct cells (middle panel), or in 8 week old sorted basal (Bas) and luminal (Lum) cells (right panel). Each are significantly downregulated in the TEB and duct cells. Their expression is restricted to the luminal cell compartment, and their downregulation in DN-Clim luminal cells suggests non-autonomous regulation of these genes by Clims through the basal cell population. Data represent mean ± SEM from at least two littermate mice. * p-value<0.05, ** p-value<0.01, *** p-value<0.001, ns: not significant.
(PDF)

Figure S5 Luminal progenitor cell analysis, representative whole mounts from DN-Clim transplants and validation of gene knockdown by siRNA. (A) CD61 was used as a marker for luminal progenitor cells in the Lin-CD29^l^°CD24^+^ population. No differences were observed in the quantity of these cells in the DN-Clim mammary gland. (B) Whole mounts of the two successful mammary transplants of DN-Clim CD29^Hi^CD24^+^ cells. Both mammary glands exhibit defects in ductal penetration and branching morphogenesis. Inset from the fat pad transplanted with 100 DN-Clim cells shows the epithelial outgrowth indicated by the arrow. (C–E) Expression of Clim1 (C), Clim2 (D), and LMO4 (E) validates specific transient knockdown of mRNA for each respective gene.
(PDF)

Figure S6 Contribution of Clim expression to prognosis prediction. Survival analysis based on expression of (A) Clim1 or (B) Clim2. Patients were divided into high and low expressing groups based on median expression of each gene. P-values derived from the Log-rank test.
(PDF)

Text S1 Supplemental materials and methods.
(DOCX)
