# Supplementary figures and images for: The Co-factor of LIM Domains (CLIM/LDB/NLI) Maintains Basal Mammary Epithelial Stem Cells and Promotes Breast Tumorigenesis
Source: PLoS Genet. 2014 Jul 31;10(7):e1004520. doi: 10.1371/journal.pgen.1004520 (PMC4117441; doi:10.1371/journal.pgen.1004520)

Figure S1

A

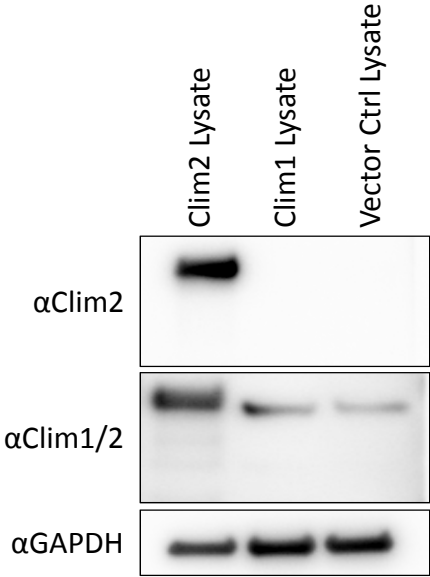

B

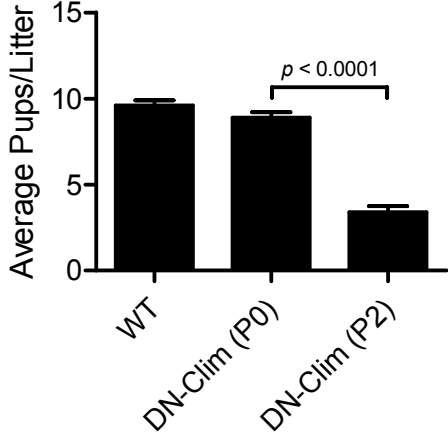

C

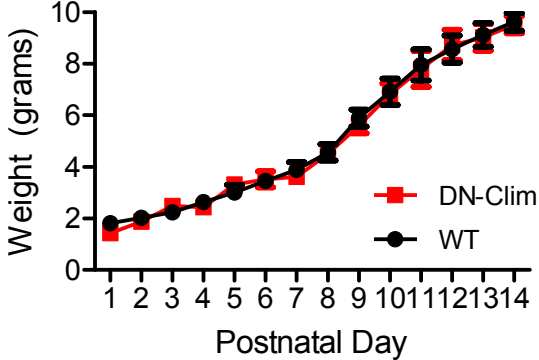

Supplement: Figure S1 — Specificity of Clim2 antibody and DN-Clim females fail to support full litters. A) The Clim2 antibody specifically targets the Clim2 protein with no reactivity to Clim1, as determined by western blot on protein lysates from HEK293 cells overexpressing the Clim1 and Clim2 proteins. The Clim1/2 antibody detects Clim1 and Clim2 only in their respective overexpression lysates. Vector Ctrl Lysate = Vector transfected lysate control. (B) Average number of pups per litter from WT and DN-Clim females. DN-Clim mice are unable to support the full litter after postnatal day 2. (C) Growth rate of pups from WT and DN-Clim females. Surviving pups from DN-Clim females grow at a normal rate compared to pups from the WT mother. (PDF) [file pgen.1004520.s008.pdf]

Figure S2

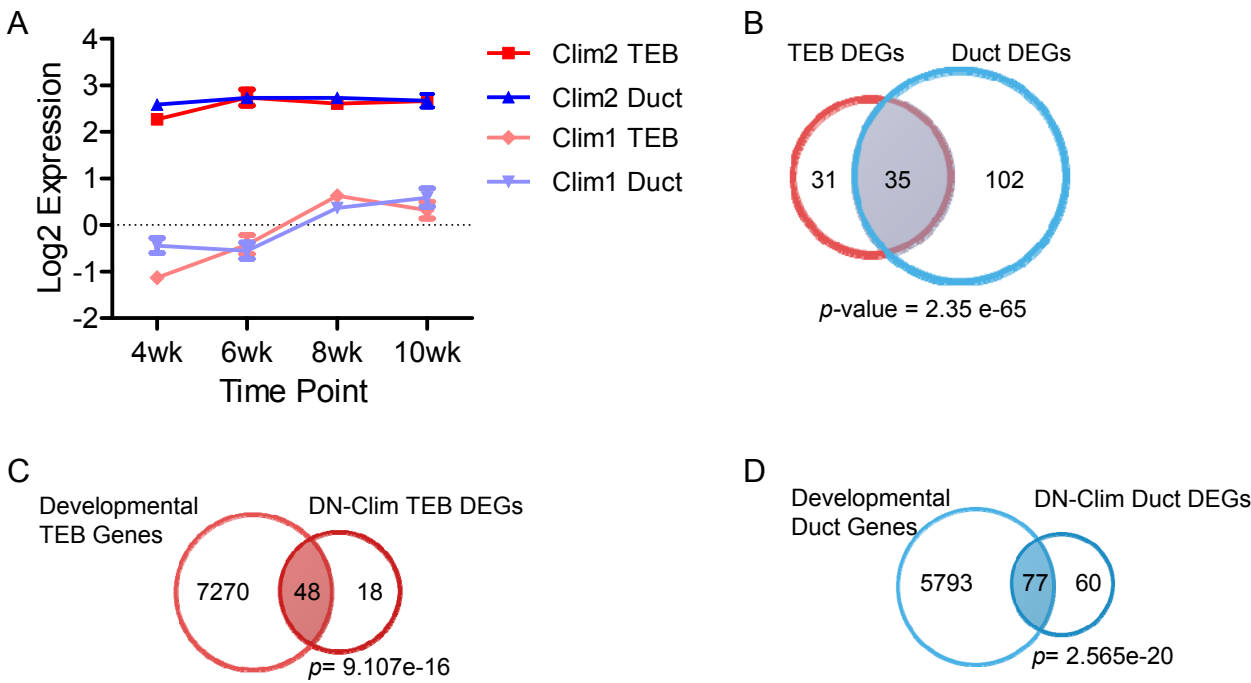

Supplement: Figure S2 — Time course analysis of Clim expression and comparison of Clim-regulated genes to TEB and duct genes. (A) Expression of Clim1 and Clim2 from time course analysis of TEB and duct cells. (B) Significant overlap of differentially expressed genes from the DN-Clim TEB and duct. (C–D) DEGs in the DN-Clim (C) TEB and (D) duct are significantly enriched in their respective developmental gene set. (PDF) [file pgen.1004520.s009.pdf]

Figure S3

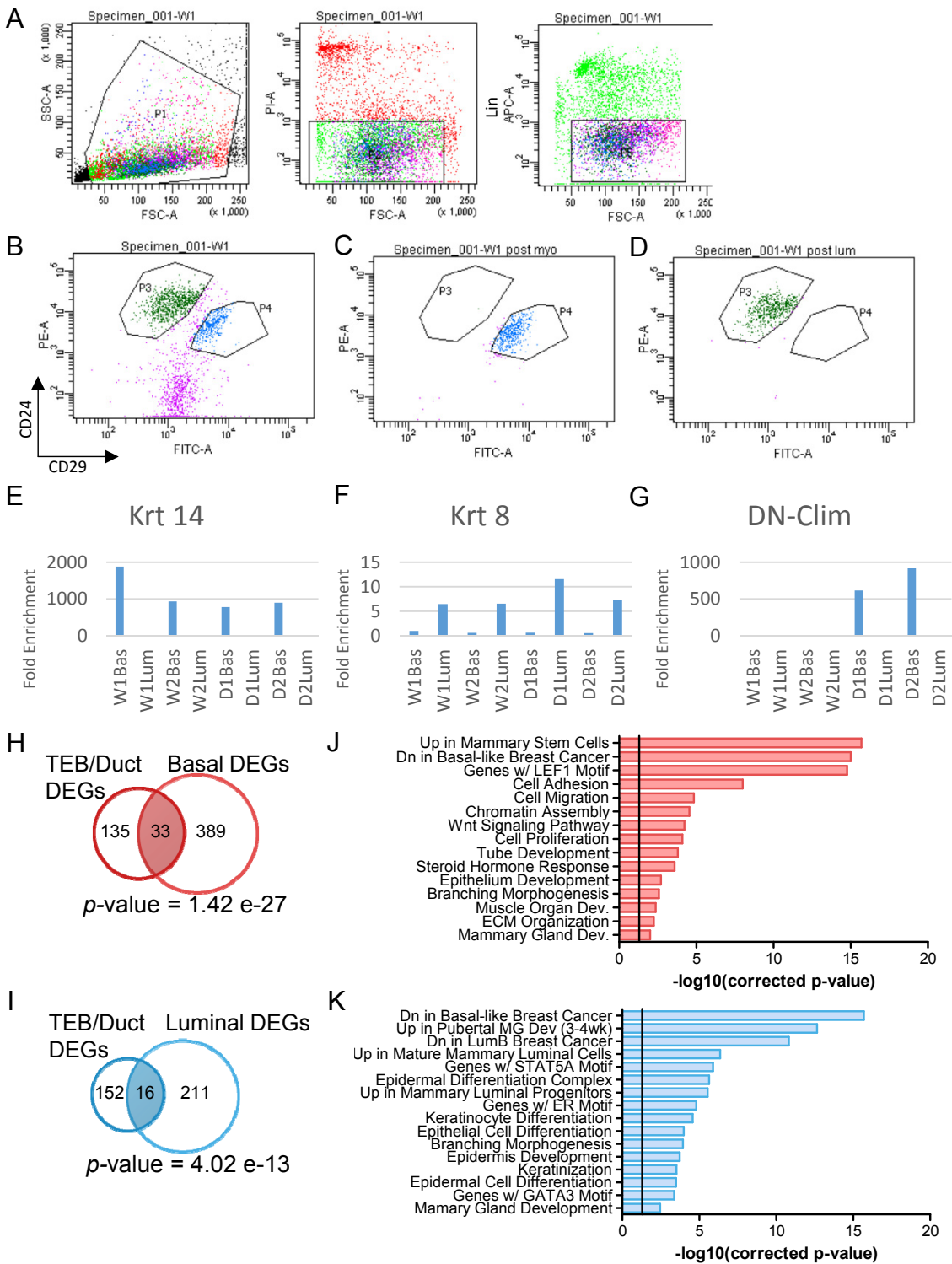

Supplement: Figure S3 — Gene expression profiling in sorted basal and luminal mammary epithelial cells. (A) Selection of live (PI-negative), Lin− (TER119-, CD45-, and CD31-negative) single cells. (B) Gating for basal (Lin−CD29HiCD24+) and luminal (Lin−CD29L°CD24+) MECs. (C) Post-sort analysis of basal MECs. (D) Post-sort analysis of luminal MECs. APC: Lin markers, PE: CD24, FITC: CD29. (E–F) qPCR validation of (E) Krt14 and (F) Krt8 in sorted cells indicates pure basal and luminal cell populations. (G) qPCR validation of DN-Clim transgene expression confirms expression of DN-Clim in basal cells. (H) DN-Clim basal and (I) DN-Clim luminal DEGs are significantly enriched in the combined list of DN-Clim TEB and Duct DEGs. Ontology analysis of (J) DN-Clim basal DEGs and (K) DN-Clim luminal DEGs. The categories represent top hits from DAVID and the Molecular Signatures Database. (PDF) [file pgen.1004520.s010.pdf]

**Figure S4**

**A**

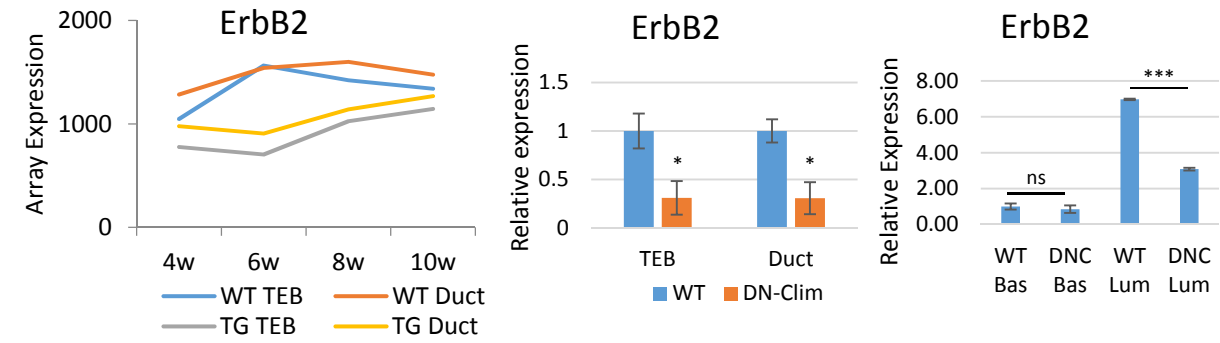

**B**

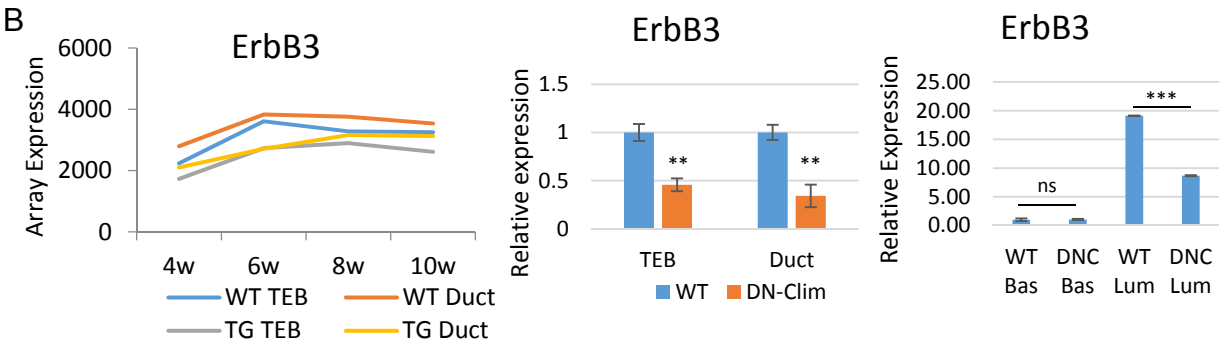

Supplement: Figure S4 — Reduced expression of ErbB2 and ErbB3 receptor tyrosine kinases in the DN-Clim mammary gland. Expression of the (A) ErbB2 and (B) ErbB3 in the time course microarray (left panel), as determined by qPCR in 6 week old laser capture microdissected TEB and duct cells (middle panel), or in 8 week old sorted basal (Bas) and luminal (Lum) cells (right panel). Each are significantly downregulated in the TEB and duct cells. Their expression is restricted to the luminal cell compartment, and their downregulation in DN-Clim luminal cells suggests non-autonomous regulation of these genes by Clims through the basal cell population. Data represent mean ± SEM from at least two littermate mice. * p-value<0.05, ** p-value<0.01, *** p-value<0.001, ns: not significant. (PDF) [file pgen.1004520.s011.pdf]

**Figure S5**

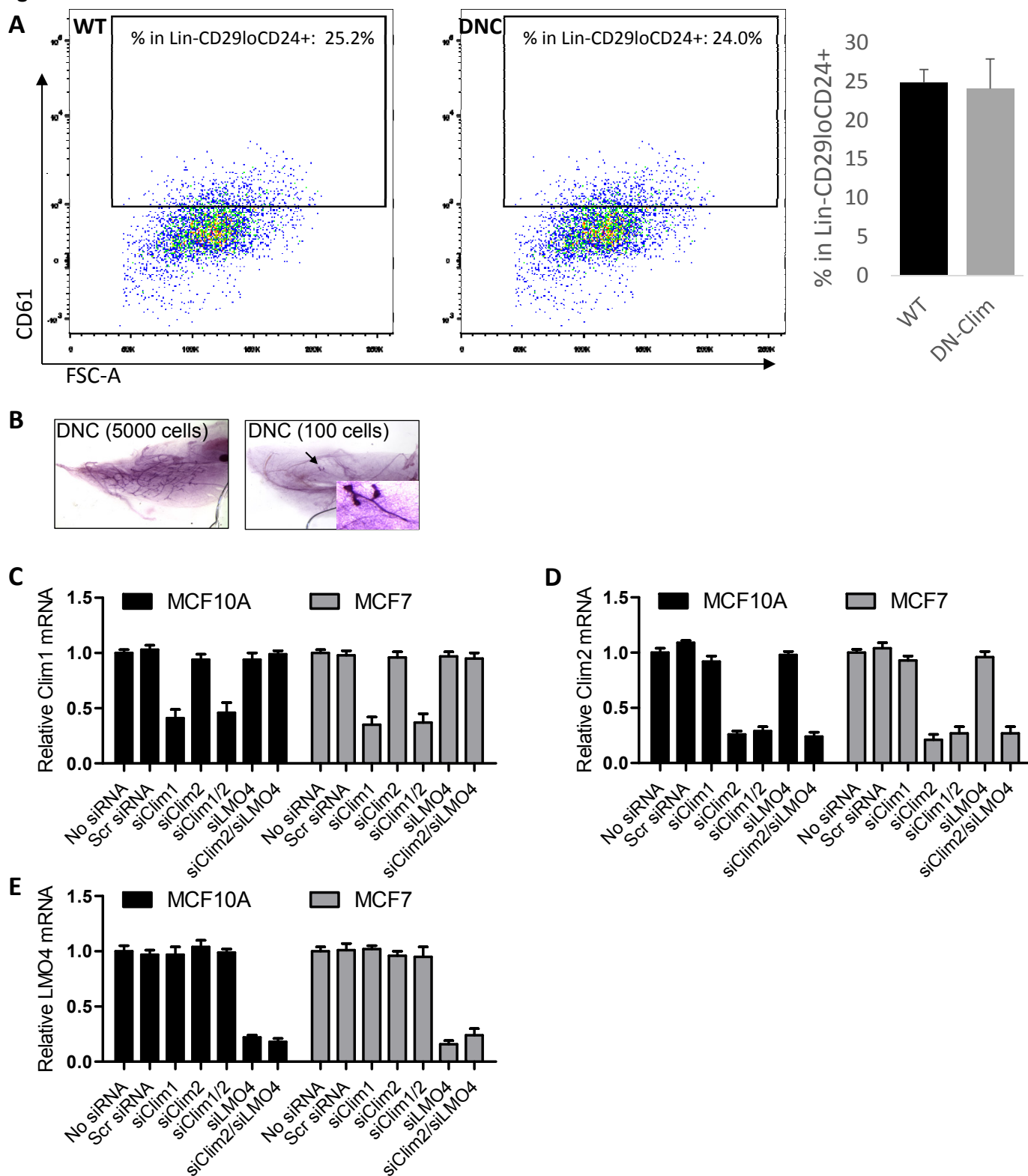

Supplement: Figure S5 — Luminal progenitor cell analysis, representative whole mounts from DN-Clim transplants and validation of gene knockdown by siRNA. (A) CD61 was used as a marker for luminal progenitor cells in the Lin-CD29l°CD24+ population. No differences were observed in the quantity of these cells in the DN-Clim mammary gland. (B) Whole mounts of the two successful mammary transplants of DN-Clim CD29HiCD24+ cells. Both mammary glands exhibit defects in ductal penetration and branching morphogenesis. Inset from the fat pad transplanted with 100 DN-Clim cells shows the epithelial outgrowth indicated by the arrow. (C–E) Expression of Clim1 (C), Clim2 (D), and LMO4 (E) validates specific transient knockdown of mRNA for each respective gene. (PDF) [file pgen.1004520.s012.pdf]

Figure S6

A

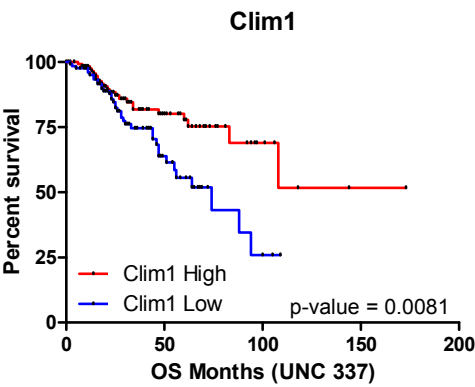

B

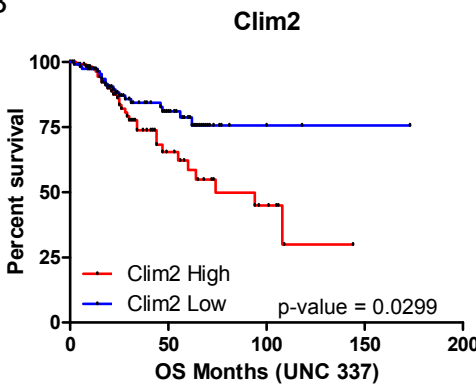

Supplement: Figure S6 — Contribution of Clim expression to prognosis prediction. Survival analysis based on expression of (A) Clim1 or (B) Clim2. Patients were divided into high and low expressing groups based on median expression of each gene. P-values derived from the Log-rank test. (PDF) [file pgen.1004520.s013.pdf]
