# Supplementary material for: The Co-factor of LIM Domains (CLIM/LDB/NLI) Maintains Basal Mammary Epithelial Stem Cells and Promotes Breast Tumorigenesis
Source: PLoS Genet. 2014 Jul 31;10(7):e1004520. doi: 10.1371/journal.pgen.1004520 (PMC4117441; doi:10.1371/journal.pgen.1004520)
Supplement: Text S1 — Supplemental materials and methods. (DOCX) [file pgen.1004520.s014.docx]

**SUPPLEMENTAL MATERIALS AND METHODS**

**Mouse strains**

*K14-DN-Clim* mice were generated in CB6F1 mice as previously described [22]. *K14-DN-Clim* males were crossed with wild type littermates, except for experiments to determine DN-Clim function in pregnancy and lactation. All experiments conform to the regulatory guidelines approved by the International Animal Care and Use Committee of the University of California, Irvine.

**Pup growth analysis**

DN-CLIM pregnant females were observed daily, and the number of living pups were counted upon birth. Pups were counted and weight was measured every other day. Analysis of differential growth rate was performed by repeated measures ANOVA.

**Whole mount preparation and analysis**

The #4 and #9 mammary glands were resected and spread on tissue specimen microscope slides (Fisher Scientific). Mammary glands with large tumors had the primary tumor resected before mounting the remaining mammary gland. Slides were fixed in Carnoy’s fixative overnight at room temperature, then washed in 70% EtOH and gradually hydrated in distilled water before staining in Carmine Alum (Sigma) overnight at 4°C. If necessary, slides were destained in 70% EtOH with 2%HCl. Slides were then rinsed in 70% EtOH and gradually dehydrated in 100% EtOH, then washed in Xylene before mounting with Permount (Fisher Scientific).

Analysis of branching morphogenesis and lobuloalveolar development was performed in ImageJ [1]. For analysis of duct length, the three longest primary ducts were measured using the lymph node as a central point of reference. At least three replicate mice were used for each time point. Statistical significance was determined by repeated measures ANOVA, and the p-value for the genotype by week interaction is reported to demonstrate a statistical difference in the rate of growth over time. Branch points were then counted for each of the three longest ducts and normalized by dividing by the length of the duct (mm). Statistical significance was determined at each time point by Student’s t-test. TEB diameter was also measured in ImageJ; at least 5 TEBs were measured per mammary gland. Statistical significance was determined in the same way as described for the duct length, and the p-value for the TEB size by week interaction is reported. Epithelial density of mammary glands from pregnant mice was also determined in ImageJ.

**Immunohistochemistry**

The #4 and #9 mammary glands were paraffin embedded and sectioned into 8µm sections. Sections were baked at 60°C for at least 30 min, then deparaffinized and hydrated by gradually washing in xylene, EtoH, water, and PBS. Antigen unmasking was performed by heating sections in 10mM sodium citrate buffer (pH 6.0) for 10 min, then blocked in dual endogenous enzyme block (Dako) and serum-free protein block (Dako). Antibodies were diluted in antibody diluent (Dako) at the following concentrations: α-Ldb1 (1:250, GeneTex 23499), α-Myc-tag (1:250, LifeTechnologies), α-BrdU (1:200, Roche), α-K8 (Troma-I, 1:500, Developmental Studies Hybridoma Bank), α-K14 (1:250, Abcam). Sections were incubated in primary antibody overnight at 4°C. Slides were incubated with the corresponding secondary antibody (1:500, Vector Laboratories) for 1hr at room temperature, then incubated in Vectastain ABC reagent (Vector Laboratories) for 1hr at room temperature, followed by treatment with DAB substrate (Vector Laboratories) and counterstained in hematoxylin. Slides were then dehydrated in gradual ethanol and xylene, then coverslips mounted with Permount. Images were taken on the Nikon Eclipse 50i and analyzed with SPOT Advanced software.

**BrdU incorporation analysis**

Mice were given intra-peritoneal injections of 5’-Bromo-2’deoxyuridine (BrdU, 50µg/g, Sigma-Aldrich) 4hrs prior to sacrificing. IHC was used to determine incorporation of BrdU into proliferating cells as described above. To quantify the percentage of BrdU+ cells, at least three mice (minimum of 5 sections from each mammary gland) were analyzed by determining the total ratio of positive to negative nuclei from at least 5 fields of view containing either TEB or duct tissue. Student’s t-test was used to determine statistical significance between genotypes.

**Immunofluorescence**

The #4 and #9 mammary glands were resected and frozen in O.C.T. compound (Fisher Scientific) and sectioned at -30°C into 6-8µm sections. Before staining, slides were warmed at room temperature for 30min, then fixed in cold acetone for 10min at -20°C. Sections were permeabilized in PBS with 0.25% TritonX for 10min, then blocked with 1%BSA in PBST for 30min. Sections were incubated in α-Fgfr2 (Bek, 1:200 Santa Cruz Biotechnology) and α-K14 (1:200, Abcam) for 1hr at room temperature, then in the corresponding secondary antibody conjugated with either AlexaFluor 488 or AlexaFluor 594 (1:1000, LifeTechnologies). Sections were then counterstained and mounted in DAPI hardmount solution (Vector Laboratories). Images were acquired on the Nikon Eclipse Ti and analyzed with NIS-Elements AR software. Apoptosis experiments were performed using the *In situ* cell death detection kit, fluorescein (Roche).

**Laser capture microdissection**

The #4 and #9 mammary glands from 4, 6, 8, and 10 week old mice were collected and frozen in O.C.T. Sections were cut at -30°C and fixed in cold acetone for 10min at -20°C, followed by 70% EtOH for 5min at room temperature. To minimize potential RNA degradation, a rapid hematoxylin and eosin staining procedure was used in which slides were washed and stained for no more than 10sec in each solution. All solutions were made with RNAse-free water. Sections were allowed to air dry for 5min prior to laser capture microdissection on the Leica LS-AMD microscope. TEB and duct tissue was microdissected in a manner to capture as much epithelial tissue as possible, minimizing the amount stroma and fat. Microdissected tissue was collected in lysis buffer with 10% 2-mercaptoethanol (Sigma-Aldrich) to preserve RNA integrity.

**RNA Isolation**

RNA from laser capture material was isolated using the RNeasy mini kit (Qiagen) according to the specifications for laser capture material. RNA from FACS sorted cells was also isolated using the RNeasy mini kit. All other RNA was isolated with TRIzol reagent (Life Technologies). When isolating RNA from mammary tissue, the lymph node of #4 and #9 mammary glands was removed. RNA concentration was measured with the NanoDrop 1000. Prior to microarray hybridization, RNA integrity was determined on the Agilent Bioanalyzer 2100.

**RT-qPCR**

First-strand cDNA synthesis for qPCR analysis was performed with iScript reverse transcriptase (Bio-Rad) and qPCR was performed with SsoFast EvaGreen Supermix (Bio-Rad) on the Bio-Rad CFX384 Real Time System. GAPDH and 18s expression were used for as controls normalization in human and mouse cells, respectively. Differential expression was determined with the ΔΔCt method. Primers sequences for each gene are as follows:

| Gene | Forward Primer | Reverse Primer |
| --- | --- | --- |
| mm18s | ATGGCCGTTCTTAGTTGGTG | GAACGCCACTTGTCCCTCTA |
| hsGAPDH | GGAGCGAGATCCCTCCAAAAT | GGCTGTTGTCATACTTCTCATGG |
| mmClim1 | ACTATCGGCAGGACCCTCATT | TGGTCGCAGTCTACTGTGATG |
| hsClim1 | ATTGGTTTTGTGGGCAGTTT | TTTAACACACGGAGCCACTG |
| mmClim2 | AAGTCATTCAAGCTGTACTCGC | TCCAGTTCTGTAGCCGTTTGT |
| hsClim2 | GGCCCAACTCCCATGTATC | TCCGGCCAATGGTATATCTC |
| mmDN-Clim | TGTATCACCATGGACCCTCAT | AAGAAGAAGGCATGAACATGG |
| hsLMO4 | GCCAGACCAGAAGGTCTGCTA | CCAATAAATGCGGGAAGGGC |
| mmKrt14 | GGACCAAGTTTGAGACGGA | ATCCCGCATCTCGTTCAG |
| mmKrt8 | GAAGTTCGTGCCCAGTACGAG | CGGTTGATGTTGCGGTTCAT |
| mmErbB2 | GCGTCCTGTTGTTCCTG | TCCAGATGCCCTTGTAGAC |
| mmErbB3 | GTGATGTCTGGAGTTACGG | CAATCATCCAACACTTGACC |
| mmFgfr2 | GATGTTGAAAGATGATGCCAC | GGTATTCCCGGAGGTTG |
| hsFgfr2 | GGAAAGTGTGGTCCCATCTGA | TCCAGGTGGTACGTGTGATTG |
| mmFgfr2 Prom (-2.0kb) | CCAGGCTGGCACTTCTATCT | GGCTTCCCTCCTATTCAGCA |
| mmFgfr2 Prom (-1.5kb) | TCAGAAGTGTGAGGCAGGAG | TCAAGCTCCCGAACTTCTCT |
| mmFgfr2 Prom (-1.0kb) | GACAGACATGCAGGCAAACA | TAGGTGGCATGGAGATCAGG |
| mmFgfr2 Prom (-0.5kb) | CTGCCATGGGAAAAGGCAAT | GGAGACCAGAGGCTTTGGAT |
| mmFgfr2 Prom (TSS) | AGTTCCTAGCCAGCTTTGGT | GCAACATCTCCCACGAACAT |
| mmFgfr2 Prom (0.5kb) | CAGAGTGGATCGGGATGGAG | CCAGTCCTTTCTTCTGCTGC |
| mmFgfr2 Prom (1.0kb) | ATTGGGATGGAGGTGCTGAA | TTCAAGGTAATCGTCGTGCC |
| mmFgfr2 Prom (1.5kb) | TGCCGTGGATTTGAAAGCAA | TCTAACTTGCGCGCTCATTC |
| mmCrisp3 (ChIP neg ctrl) | TCACTATGGGCAGAAATGTTG | TGAGCAAATTCAAGGAAGCA |

**Mouse cell preparation, flow cytometry, and sorting**

Adult (8 to 12 week) mammary glands for single-cell suspensions were generated according to Stem Cell Technologies protocol. When collecting RNA from cells, the collagenase/hyaluronidase digestion was reduced to 1.5 hours. For sorting and flow cytometry, the single cell suspensions were labeled with CD31-APC, CD45-APC, TER119-APC, CD24-PE or CD24-PE-Cy7, CD29-FITC, and CD61-PE antibody in PBS+2%FBS for 30 minutes at room temperature. Cells were washed with PBS+2%FBS and labeled with Propidium iodide before analysis. Flow cytometry analysis was performed on the BD FACSCalibur and sorting was performed on the BD FACSAriaII. Live, single cells were gated to exclude lineage cells labeled with CD31, CD45, and TER119. For sorted cells, immediate post sorting analysis indicate at least 98% purity of cell populations

**Mammary stem cell transplants into cleared mammary fat pads**

For limiting dilution transplantation, sorted basal cells of each genotype were counted and

resuspended in a 1:1 solution of PBS/Matrigel (BD Biosciences) at desired concentrations

(5,000, 1,000, 500, 100, and 50 cells/10 µL). Cell/Matrigel solutions were then injected in a 10-µL volume into contralateral cleared fat pads of inguinal mammary glands of 3-week-old female SCID/Beige mice (Charles River) using a custom ordered 26-G needle attached to a 50-µL Hamilton glass syringe. Mice were anesthetized by an intraperitoneal injection of ketamine and xylazine (750 and 50 mg per kg body weight, respectively). Outgrowths were analyzed eight weeks after transplantation. Statistical analysis of the take rate was performed using the ELDA Web-based tool (<http://bioinf.wehi.edu.au/software/elda/>) [2].

**Mammary colony forming and mammosphere culture**

When culturing primary murine mammary epithelial cells, single cell suspensions were enriched for mammary epithelial cells by immunolabeling with biotinylated lineage markers and magnetic cell separation (Stem Cell Technologies). For colony forming cell assays, single cells were seeded at a density of 250 cells/cm^2^ onto plates with Mitomycin C (10µg/mL) treated NIH 3T3 feeder cells (1000 cells/cm^2^) and cultured in EpiCult-B Medium per manufacturer’s instructions (Stem Cell Technologies). After seven days of culture, colonies were stained with Wright-Giemsa (Sigma-Aldrich) and counted. Colony size was measured in ImageJ.

For mammosphere assays, 10,000 cells per well were seeded into 24-well ultra-low attachment plates (Fisher Scientific). Primary mouse cells were grown in complete EpiCult B medium supplemented with B27 (Life Technologies). MCF10A and MCF7 cells were grown in DMEM/F12 supplemented with B27, EGF (20ng/mL), bFGF (20ng/mL), Heparin (4µg/mL), and 2%FBS. Mammospheres were counted after 7 days. When passaging, mammospheres were collected by centrifugation, then dissociated in 0.05% Trypsin by gentle pipetting. Cells were then passed through 40µm cell strainer to remove any extra aggregates. 5,000 cells were re-seeded into each well.

**Transfection and lentiviral expression**

MCF10A and MCF7 cells were transfected with Clim1, Clim2, or LMO4 siRNAs (Qiagen) using Lipofectamine RNAiMAX transfection reagent (Life Technologies). Three siRNAs per gene were optimized and validated individually for optimal transfection conditions and specificity of gene knockdown. For mammosphere assays the siRNAs were pooled and reverse transfected into cells on adherent plates. The following day, transfected cells were seeded for mammosphere assays. RNA was collected before seeding for mammosphere assays and after counting mammospheres to validate continued mRNA knockdown throughout the assay.

For lentiviral expression in MCF10A cells, GFP and Fgfr2 expression constructs (GeneCopoeia) were transfected into the 293Ta lentiviral packaging cell line (GeneCopoeia) per manufacturer’s instructions. The Lenti-Pac HIV Expression packaging kit (GeneCopoeia) was used to generate lentiviral particles. Viral particles were used to transduce MCF10A cells in complete growth medium and Ploybrene (5µg/mL). Successfully transduced cells were selected by treating with Puromycin every 3-4 days. Expression of GFP was validated by visually inspecting fluorescence, and expression of Fgfr2 was validated by western blot.

**Chromatin immunoprecipitation and luciferase assays for Fgfr2 promoter analysis**

For chromatin immunoprecipitation assays, primary mammary epithelial cells were collected and pooled from 4 wild type and 4 DN-Clim mice. During mammary cell preparation, Collagenase/Hyaluronidase treatment was reduced to 1hr. Fat was removed by centrifugation and the remaining pellet was treated with ammonium chloride to remove erythroid cells.

ChIP assays were performed as previously described [3]. Protein-DNA complexes were crosslinked by treating with 1% Formaldehyde in DPBS with protease inhibitors (PMSF (1mM), Aprotinin (1µg/mL), and Leupeptin (1µg/mL)) and incubating 15min at room temperature with gentle shaking. Crosslinking was stopped by adding glycine to 0.125M final concentration and incubating for 5min at room temperature with gentle shaking. Protein was collected and sonicated (15s on/off cycle for 45 min).

ChIP was performed with Dynabeads (Life Technologies). Dynabeads were incubated overnight at 4°C with 2µg antibody (IgG (Sigma), Clim2 (Stem Cell Technologies), LMO4 (Stem Cell Technologies), Myc-tag (Life Technologies), or H3K4me3 (Millipore)). IP was performed by adding 24µg of sonicated chromatin to each antibody/bead complex and incubating at 4°C overnight on a rotating platform. Protein-DNA complexes were reverse crosslinked by adding NaCl to 0.2M final concentration and incubating overnight at 67°C. Enrichment of target DNA sequences was analyzed by qPCR and calculated as fold enrichment over IgG. Crisp3, a gene that is not expressed in the mammary gland, was used as a negative control.

The Clim/LMO4 binding region upstream of the *Fgfr2* TSS in the mouse was cloned using the following primers targeting the promoter sequence 433 to 1,010 base pairs upstream of the *Fgfr2* TSS: Forward – CTCCTGCCTCACACTTCTGA, Reverse – AATTCCCAGCATCCACTTGG. The NheI and XhoI restriction enzyme sites were added to the 5’ end of the forward and reverse primers, respectively. The DNA fragment was inserted into the pGL3-promoter luciferase reporter vector (Promega). The reporter vector, DN-Clim expression vector, and renilla luciferase control vector were transfected into MCF10A cells using X-tremeGENE HP transfection reagent (Roche). The Dual Luciferase Reporter assay (Promega) was used to measure luciferase per manufacturer’s instructions.

**MMTV-PyMT breast tumor analysis**

For analysis of tumor-free survival, mice were monitored daily until the first palpable tumor is observed. From that time, calipers were used to measure length and width of tumors every other day for 14 days and volume was estimated with the following formula: (π/6)*(length^2^)*width. Growth rate of the first and second observed tumors were analyzed for statistical differences by repeated measures ANOVA. The p-value presented represents the significance when comparing growth rate (the interaction between tumor volume and time).

**Microarray analysis**

RNA for gene expression profiling was analyzed for integrity on the Agilent Bioanalyzer 2100. For laser capture microdissected material, RNA Integrity Number (RIN) was at least 8 for each sample, and FACS sorted cells had RINs of at least 9.5. For the timecourse analysis of TEB and duct cells we used only littermate mice for each time point, resulting in 3 WT and 3 DN-Clim replicates for the 4 week time point, 3 WT and 2 DN-Clim 6 week old replicates, 2 WT and 3 DN-Clim 8 week old replicates, and 2 WT and 3 DN-Clim 10 week old replicates. Arrays were processed at the UC Irvine Genomics High Throughput Facility, and analyzed by PLIER analysis. Genes with raw expression values below 200 in every sample were classified as non-expressed and removed from analysis. The data was log2 transformed and mean centered with unit variance. To determine genes that are developmentally regulated in each WT cell type over the time course, we applied the Bayesian Estimation of Temporal Regulation algorithm [24], using a p-value cutoff of 0.01. Average fold change between TEB and duct for each gene was calculated for each time point, and developmentally regulated genes (BETR p-value <0.01) with more than 1.5-fold change in at least two time points were used to classify gene signatures for TEB and duct cells. To determine differentially expressed genes in the DN-Clim mammary gland we applied the CyberT algorithm to compare expression in WT and DN-Clim TEB and WT and DN-Clim duct cells [4]. Genes with p-value < 0.001 and at least 1.5-fold change in at least two time points were classified as differentially expressed. Gene modules were analyzed for enrichment of functional categories and other gene signatures using DAVID [5] and the Molecular Signatures Database [32]. For comparison of overlap between gene modules, the hypergeometric test was applied, using the total number of genes reported for the Affymetrix Mouse Gene 1.0ST as the population size.

For gene expression profiling of FACS sorted basal and luminal cells, 2 WT and 2 DN-Clim littermates were used as replicates. Arrays were processed in the same manner as described above, with the exception that genes with raw expression values below 150 in all samples were removed from the analysis. Differential expression between WT and DN-Clim basal, WT and DN-Clim luminal, and WT basal and luminal cells was determined with the CyberT algorithm. Genes with a p-value < 0.01 and fold change of at least 1.5 were classified as differentially expressed.

Expression of gene sets were analyzed in three independent breast cancer studies with gene expression microarray and clinical survival data: UNC337 [27], NKI295 [28], and CRI537 [29]. The RMA algorithm followed by mean centering and conversion to unit variance was used to process each data set. Mouse gene identifiers were converted to their human homologs, and genes for each module were extracted; genes with multiple probes were averaged. Statistical comparison of the expression of gene modules in the different subtypes of breast cancer was performed by ANOVA. For survival analysis, the average expression of each gene module was calculated for each tumor sample, then each data set was split into high and low expressing groups based on median expression of these values. Proliferation genes were removed from gene signatures as described by Ben-Porath et al [6]. Statistical comparison of survival curves was performed with the Log-rank test and hazard ratios with 95% confidence intervals are reported.

**REFERENCES FROM TEXT S1**

1. Schneider CA, Rasband WS, Eliceiri KW (2012) NIH Image to ImageJ: 25 years of image analysis. Nat Methods 9: 671-675.

2. Hu Y, Smyth GK (2009) ELDA: extreme limiting dilution analysis for comparing depleted and enriched populations in stem cell and other assays. J Immunol Methods 347: 70-78.

3. Yu Z, Mannik J, Soto A, Lin KK, Andersen B (2009) The epidermal differentiation-associated Grainyhead gene Get1/Grhl3 also regulates urothelial differentiation. EMBO J 28: 1890-1903.

4. Kayala MA, Baldi P (2012) Cyber-T web server: differential analysis of high-throughput data. Nucleic Acids Res 40: W553-559.

5. Dennis G, Sherman BT, Hosack DA, Yang J, Gao W, et al. (2003) DAVID: Database for Annotation, Visualization, and Integrated Discovery. Genome Biol 4: P3.

6. Ben-Porath I, Thomson MW, Carey VJ, Ge R, Bell GW, et al. (2008) An embryonic stem cell-like gene expression signature in poorly differentiated aggressive human tumors. Nat Genet 40: 499-507.
